# SARS-CoV-2 Infections in the World: An Estimation of the Infected Population and a Measure of How Higher Detection Rates Save Lives

**DOI:** 10.3389/fpubh.2020.00489

**Published:** 2020-09-25

**Authors:** Carlos Villalobos

**Affiliations:** Escuela de Ingeniería Comercial, Centro de Investigación en Economía Aplicada, Facultad de Economía y Negocios, Universidad de Talca, Talca, Chile

**Keywords:** infection fatality ratio, infection detection ratio, estimates of SARS-CoV-2 infections, asymptomatic SARS-CoV-2 population, multiple linear regression

## Abstract

This paper provides an estimation of the accumulated detection rates and the accumulated number of infected individuals by the novel severe acute respiratory syndrome coronavirus 2 (SARS-CoV-2). Worldwide, on July 20, it has been estimated above 160 million individuals infected by SARS-CoV-2. Moreover, it is found that only about 1 out of 11 infected individuals are detected. In an information context in which population-based seroepidemiological studies are not frequently available, this study shows a parsimonious alternative to provide estimates of the number of SARS-CoV-2 infected individuals. By comparing our estimates with those provided by the population-based seroepidemiological ENE-COVID study in Spain, we confirm the utility of our approach. Then, using a cross-country regression, we investigated if differences in detection rates are associated with differences in the cumulative number of deaths. The hypothesis investigated in this study is that higher levels of detection of SARS-CoV-2 infections can reduce the risk exposure of the susceptible population with a relatively higher risk of death. Our results show that, on average, detecting 5 instead of 35 percent of the infections is associated with multiplying the number of deaths by a factor of about 6. Using this result, we estimated that 120 days after the pandemic outbreak, if the US would have tested with the same intensity as South Korea, about 85,000 out of their 126,000 reported deaths could have been avoided.

## Introduction

Governments and policymakers dealing with the COVID-19 pandemic will fail in their objectives if their actions are guided by misleading data or subsequent misinformation. The authorities should have reliable estimations of the number of SARS-CoV-2 infected individuals. However, there are few attempts to estimate the total amount of infections (1–5). Consequently, health systems face enormous challenges since an unknown and probably a high proportion of all SARS-CoV-2 infections remains undetected. Moreover, data suggest that infected individuals can be highly contagious before the onset of symptoms and SARS-CoV-2 can be also highly contagious in individuals who will never develop any symptoms (6–10).

Undetected infections are dangerous because infectious individuals spread the coronavirus in unpredictable ways. Undetected infections consist of non-PCR-tested individuals with symptoms and asymptomatic individuals (non-COVID-19 patients) that are likely to remain undetected over all phases of the infection. However, non-PCR-tested individuals with symptoms would tend to auto-select themselves, depending on the severity of their symptoms (from mild to severe), toward treatment and late detection. For this reason, it is important to know the proportion of the infected population which is asymptomatic or has such mild symptoms that self-select them into the group of non-PCR-tested individuals (11–15). Here, regarding the estimation of the number of infections, and for purposes of public health, I advocate the view by Amartya Sen and Martha Nussbaum that is preferable to be vaguely right than precisely wrong.

The public health problem is that undetected asymptomatic individuals, as well as late-detected SARS-CoV-2 infected individuals, increase the risk for vulnerable groups^1^. Since there is a transmission channel between the level of detection and the number of deaths, the early detection of asymptomatic infections, pre-symptomatic, and mild COVID-19 cases is a public health concern.

Moreover, undetected cases also are responsible for the collapse of the health system by numerous aggravated and sometimes unexpected COVID-19 patients requiring treatment in a short period. Overwhelmed health care systems reduce the recovery prospects of patients by the lack of treatment, undertreatment, increased risk of mistreatment of all patients, including those with COVID-19, and also put at unnecessarily risk the health workforce (21, 22).

The problem is that many governments formulate their strategies and responses to the pandemic based on figures that they can control. This problem of reverse causality produces contra-productive incentives for governments since public opinion tends to negatively react to the report of the cumulative and the marginal numbers of detected (reported) cases. The contradiction is that something good, such as the increase in the testing efforts by governments can be perceived by the public opinion as something bad (due to the increase in detections). Worldwide, the media communicates confirmed cases and deaths as the relevant parameters to take into consideration when assessing the evolution of the pandemic. This is a mistake since this emphasis discourages governments from decidedly pushing for mass testing with the obvious consequence of an increased number of detected cases (although, as shown in this paper, there is a theoretical mechanism relating more testing with saving lives). More sophisticated observers would use the crude and adjusted case fatality ratios to assess the pandemic evolution. However, international comparisons show that crude and adjusted case fatality ratios are highly heterogeneous and their use can be misleading (23, 24). For instance, the simple division of the cumulative number of deaths by the cumulative number of confirmed cases underestimated the true case fatality ratio in past epidemics (24, 25). Although nowadays many case fatality ratios have been estimated in this pandemic correcting many of the observed past biases (26–28), they are still depending on testing efforts made by countries.

The problem with heterogeneous case fatality ratios (different proportions of all cases that will end in death due to methodological differences on the denominator) is that they are not anchored at any exogenous information that allows researchers to perform international or territorial comparisons based on credible, and transparent assumptions. Consequently, to rely on the number of confirmed cases makes international comparations impossible since governments have shown to implement highly heterogeneous SARS-CoV-2 testing strategies ending up in different levels of location-based under-ascertainment.

In an attempt to solve the mentioned problem, we anchor our analysis in the cumulative number of deaths, which is a statistic much more difficult to alter, in free societies, than the number of SARS-CoV-2 tests^2^.

We use this information together with the newest and sound estimates of the age-stratified infection fatality ratios (IFRs) provided in the recent SARS-CoV-2 related literature. In particular, we base our analysis on the IFR of 0.657% reported in Verity et al. (26). This IFR is very close to the 0.75% reported in a meta-analysis of 13 IFR estimates from a wide range of countries, and that were published between February and April of 2020 (30). We also assume orthogonal attack rates of the infection which is also supported by recent literature (16). By weighting the age-stratified IFRs by the country population age-groups shares in each country, it is possible to obtain country-specific IFRs.

The relevance of this study is 3-fold: Firstly, the estimation of the true number of infections includes not only confirmed cases but COVID-19 undetected cases, as well as SARS-CoV-2-infected individuals without the disease, or in a pre-symptomatic stage. Therefore, to provide an estimation of the true number of SARS-CoV-2 infections is of more utility than to be only informed about the number of confirmed infections. This is because confirmed cases depend on the testing efforts that can be altered or even manipulated by governments. Moreover, one can compare the true estimate of infections with the number of COVID-19 patients that require hospitalization. Such ratios can contribute to predicting, with exogenous-to-government information, shortages of the health systems. Secondly, the estimation of the true number of SARS-CoV-2 infections allows us to estimate the detection rate of the infection, which is a measure of the performance of health systems and governments while facing the pandemic. One can expect that higher levels of detection of SARS-CoV-2 infections, which includes asymptomatic population, and those in their early stages of the infection (which are more infectious) can reduce the risk exposure of the susceptible population with relatively a high risk of death, that is, the elderly and those individuals with preexisting conditions (17). Accordingly, a highly neglected statistic, such as the detection rate should be considered highly relevant from the public health point of view. Thirdly, in this paper, we test the hypothesis that higher detection rates can save lives while providing a measure of this impact (having in mind that is preferable to be vaguely right than precisely wrong). Thus, this study aims to quantify the importance of testing while providing empirical support to the utility of implementing massive SARS-CoV-2 tests.

Overall, this study argues that it is crucial to compute the evolution of the cumulative number of estimated SARS-CoV-2 infected individuals, and subsequently, the cumulative detection rates. This information would provide public health managers and governments the incentives to improve detection rates, rather than to the opposite. Moreover, the identification strategy can be used at lower levels of aggregation, such as regions, provinces, and municipalities to improve responses to the pandemic, including the planning of selective lockdowns or spatial-selective enhancements of the installed critical care units.

In summary, this study proposes a baseline estimation of the number of SARS-CoV-2 infections and detection rates based on current information and transparent assumptions. However, the assumptions discussed later in this paper can be later modified to match the current scientific available evidence and country-specific developments and contexts.

## Data and Methods

### Data

For this research, we use the cumulative number of deaths and confirmed cases in the world and by country, published by OurWorldInData.org, a project of the Global Change Data Lab with the collaboration of the Oxford Martin Programme on Global Development at the University of Oxford^3^. Age-stratified demographic proportions of the population were obtained from the UN population data^4^. The age-stratified IFRs are those reported in Verity et al. (26)^5^. Our method also requires to know the distribution of the number of days between infection and death. Since this number is unknown, we approach to this number using the sum of the median incubation period as reported in Lauer et al. (31), and the mean number of days between the onset of symptoms and death as reported in Verity et al. (26). For our empirical exercise, we rely on World Development data by the World Bank (GDP per capita and health expenditure as a share of the GDP)^6^ and in World Health Organization data for BCG vaccination^7^.

In this study, our regression analysis relies on data for 91 countries covering above 86% of the world population. The remaining countries were excluded because they either do not have significant mortality figures (for instance Uruguay, Monaco, Bermuda, etc.), or full data.

### Methods

#### Estimation Strategy

In this study, we rely on a very simple rationale. At a given point in time, the cumulative number of deaths should be a proportion of the cumulative number of infections somewhat in the past. But how many days in the past? The answer lies in the sum of the number of days of incubation and the number of days between the onset of symptoms and death. This rationale follows a report focusing on the 40 most-affected countries by the pandemic in the world (32). However, in this paper, we deviated from the mentioned report by using the key parameters in a different way, which translated into a different estimation of the number of infected individuals.

On average, deaths occur ~18 days (17.8 days with 95% credible interval [CrI] 16.9–19.2) after the onset of COVID-19 symptoms (26), while the incubation period of COVID-19 has been estimated in about 5 days (5.1 days with 95% CI, 4.5–5.8) as reported in Lauer et al. (31). Thus, by comparing the cumulative number of deaths at time *t* in country *i* (*cdeaths*_(*i, t*)_) with the country-specific infection fatality ratio (*ifr*_*i*_), which is assumed constant over time, it is possible to obtain a rough approximation of the cumulative number of SARS-CoV-2 infections 23 days (18 days + 5 days) in the past (*cinfected*_(*i*,*t*_−23_)_)^8^.

(1) $$\begin{array}{l} cinfected_{\left(i,t_{-23}\right)}=\frac{cdeaths_{\left(i,t\right)}}{\;ifr_{i}} \end{array}$$

Additionally, we use the ratio between the cumulative number confirmed (detected) cases at time *t*_−23_ in country *i* (*cconfirmed*_(*i*,*t*_−23_)_) and the cumulative number of infected individuals (*cinfected*_(*i*,*t*_−23_)_) at time *t*_−23_ in country *i* as a rough measure of the cumulative rate of detection of SARS-CoV-2 infections at time *t*_−23_.

(2) $$\begin{array}{l} {detection\;rate}_{\left(i,t_{-23}\right)}=\frac{cconfirmed_{\left(i,t_{-23}\right)}}{\;cinfected_{\left(i,t_{-23}\right)}} \end{array}$$

#### Infection Fatality Ratio

In order to estimate the country-specific infection fatality ratio for country *i* used in equation 1, we weight the age-stratified infection fatality ratios reported in Verity et al. (26), by the age-group population shares of country *i*. The calculation of the age-stratified infection fatality ratios relies on two assumptions that can be modified when producing point estimates of the number of individuals affected by a SARS-CoV-2 infection. Firstly, it assumes that there are no cross-country differences in the average overall health status of the population, comorbidity, or in the soundness of the different health systems. In absence of standardized country-specific information of these variables, this assumption is convenient although, at first sight, it can be considered a restrictive one. However, it is quite the opposite since, in richer countries with higher proportions of elderly populations, the estimated infection mortality ratios are likely to be overestimated. If so, our estimates of the infected population represent a lower limit of the true number of infections. The second assumption is that the attack rate of the coronavirus is unrelated to the age and sex of susceptible individuals. This is in concordance with the evidence in respiratory infections in previous pandemic processes (26, 33). Then, the distribution of IFRs across countries reflects the “fixed” lethality of the virus associated to a varying demographic structure of the population across the world.

**Figure 1:**
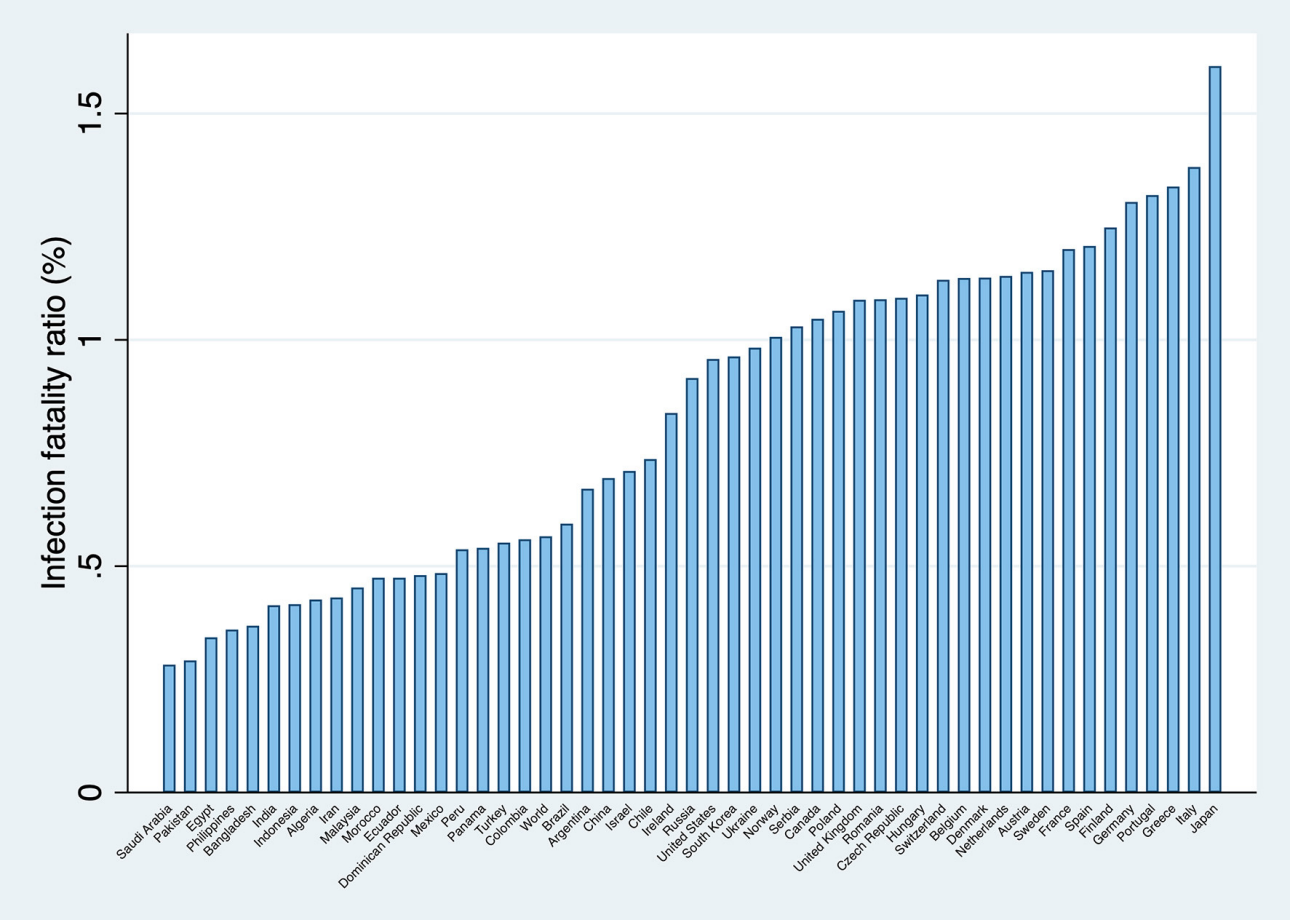
Infection fatality ratios (selected countries, in percentage). Source: Own elaboration.

Figure 1 presents the calculated infection fatality ratios for the world, and for 50 countries in which the lethality of the pandemic has been more significant.

Recently, a cross-sectional epidemiological study with a super-spreading event in the county of Heinsberg in Germany offered the opportunity to estimate the infection fatality ratio in the community (34). The estimated infection fatality ratio was 0.36%. Although this number is surprisingly low when compared with other estimations, for instance, the used in this study for Germany (1.3%), it is not evident that the true infection fatality ratio is closer to 0.36% rather than 1.3%. This is because there can be local factors that explain the discrepancy as pointed out in the Heinsberg study. Amongst these factors, it might be mentioned comorbidity gaps, ethnic differences, the quality and coverage of the health systems, climatic differences, immunization levels, etc.^9^.

Consequently, it might be necessary to assess the consequences of using an overestimated infection fatality ratio (that is, an IFR closer to the one reported in the Heinsberg study, or others inferred from seroprevalence data (36). The answer is that the number of infections would be underestimated, and that detection rates would be overestimated (since the infection fatality ratio is on the denominator). An overestimation of the detection rates reduces the validity of international rankings based on this figure. However, from the public health point of view, this would be irrelevant since, as discussed later, all countries should increase their detection rates of SARS-CoV-2 infections as much as possible.

#### Regression Analysis

To investigate whether improving the detection rates of SARS-CoV-2 infections is potentially associated to save lives, we use a parsimonious synchronic cross-country multiple linear regression^10^. That is, we use the information reported 15, 60, and 105 days after the confirmation of the first 100 SARS-CoV-2 infections, which corresponds to the pandemic outbreak (PO). At a given pandemic phase, we regress the natural logarithm of the cumulative number of deaths in country *i*, ln(*deaths*_*i*_), on their estimated detection rates (*DR*_*i*_) and its squared to assess whether there is a non-linear relationship of this conditional correlation^11^.

The four parsimonious regressions have a demographic control that corresponds to the estimated country-specific infection fatality ratio (*ifr*_*i*_). This is a non-endogenous control since it only captures the impact of demography (population shares by age-groups) on the number of deaths and not the reverse. The regressions control for the population size of the country *i* in its natural logarithmic form ln(*pop*_*i*_). This control is necessary because the share of the susceptible population remains persistently at relatively higher levels in more populated countries when compared with the less populated ones. We also include the natural logarithm of the number of confirmed SARS-CoV-2 infections in each country ln(*confirmed*_*i*_). This is a measure of the persistence of the mortality process while controlling for cross-country differences in their absolute testing performances. The regressions also control for the economic performance of a country by means of the natural logarithm of the per capita gross domestic product ln(*gdppc*_*i*_)^12^. We also include the current health expenditure as share of GDP in 2017 (*healthshare*_*i*_). This control is needed to account for relative resource-dependent differences in the coverage/quality of the health systems around the globe. Finally, we use available data to explore a possible association between BCG vaccination and aggravated cases of COVID-19, and deaths [a relationship which is being investigated in some clinical trials (37)]^13^. The evidence is still inconclusive because the argued existence of uncontrolled confounders (38–42). However, if these confounders exist, they can bias the relationship between SARS-CoV-2 detections rates and the cumulative number of deaths. Based on this argument, we include a raw of dummies capturing the degree of BCG vaccination coverage as follows: *BGC group 1*: no mandatory vaccination (up to 49.9% coverage), *BGC group 2*: 50 to 79.9% coverage, *BGC group 3*: 80 to 89.9%, *BGC group 4*: 90 to 98.9%, and *BGC group 5*: 99 to 100%. The reference category is *BCG group 1*.

(3) $$\begin{array}{l} \ln\left(deaths_{i}\right)=\alpha +\beta_{1}DR_{i}+\beta_{2}DR_{i}^{2}+\beta_{3}ifr_{i}+\beta_{4}\ln(pop_{i})\\ \;+\beta_{5}\ln(confirmed_{i})+\;\beta_{6}\ln\left(gdppc_{i}\right)\\ \;+\beta_{7}healthshare_{i}+\beta_{8}bcg_{2i}+\beta_{9}bcg_{3i}+\beta_{10}bcg_{4i}\\ \;+\beta_{11}bcg_{5i}+\mu_{i}\;\forall \;i=1,\ldots ,91 \end{array}$$

#### Robustness

An alternative approach is used to indirectly investigate the conditional association between detection rates and SARS-CoV-2 related deaths. Instead of using the detection rates and its square, we use the natural logarithm of the estimated number of infections ln(*infections*_*i*_) while dropping from the equation the natural logarithm of the number of confirmed (detected) SARS-CoV-2 infections as follows:

(4) $$\begin{array}{l} \ln\left(deaths_{i}\right)=\alpha +\beta_{1}\ln(infections_{i})+\beta_{2}ifr_{i}+\beta_{3}\ln(pop_{i})\\ \;+\;\beta_{4}\ln(gdppc_{i})+\beta_{5}healthshare_{i}\;\\ \;+\beta_{6}bcg_{2i}+\beta_{7}bcg_{3i}+\beta_{8}bcg_{4i}+\beta_{9}bcg_{5i}+\mu_{i}\\ \text{ ​​​​​​}\forall \;i=1,\ldots ,91 \end{array}$$

Regarding the statistical inference, significance tests rely on a heteroscedasticity consistent covariance matrix (HCCM) type HC3 which is suitable when the number of observations is small (43). Although in the presence of heteroscedasticity of unknown form, Ordinary Least Square estimates are unbiased, the inference can be misleading due to the fact that the usual tests of significance are generally inappropriate (43).

Additionally, we estimate the same set of equations (the main specification and the robustness specification 15, 60, and 105 days after the pandemic outbreak) using robust regressions. We do this because we have the concern that parameter estimates may be biased if, in some countries (outliers), the report of the cumulative number of deaths has been involuntarily altered or even manipulated. Robust regression resists the effect of such outliers, providing better than OLS efficiency when heavy-tailored error distributions exist as it can be likely the case (44).

## Results

### Descriptive Analysis

**Figure 2:**
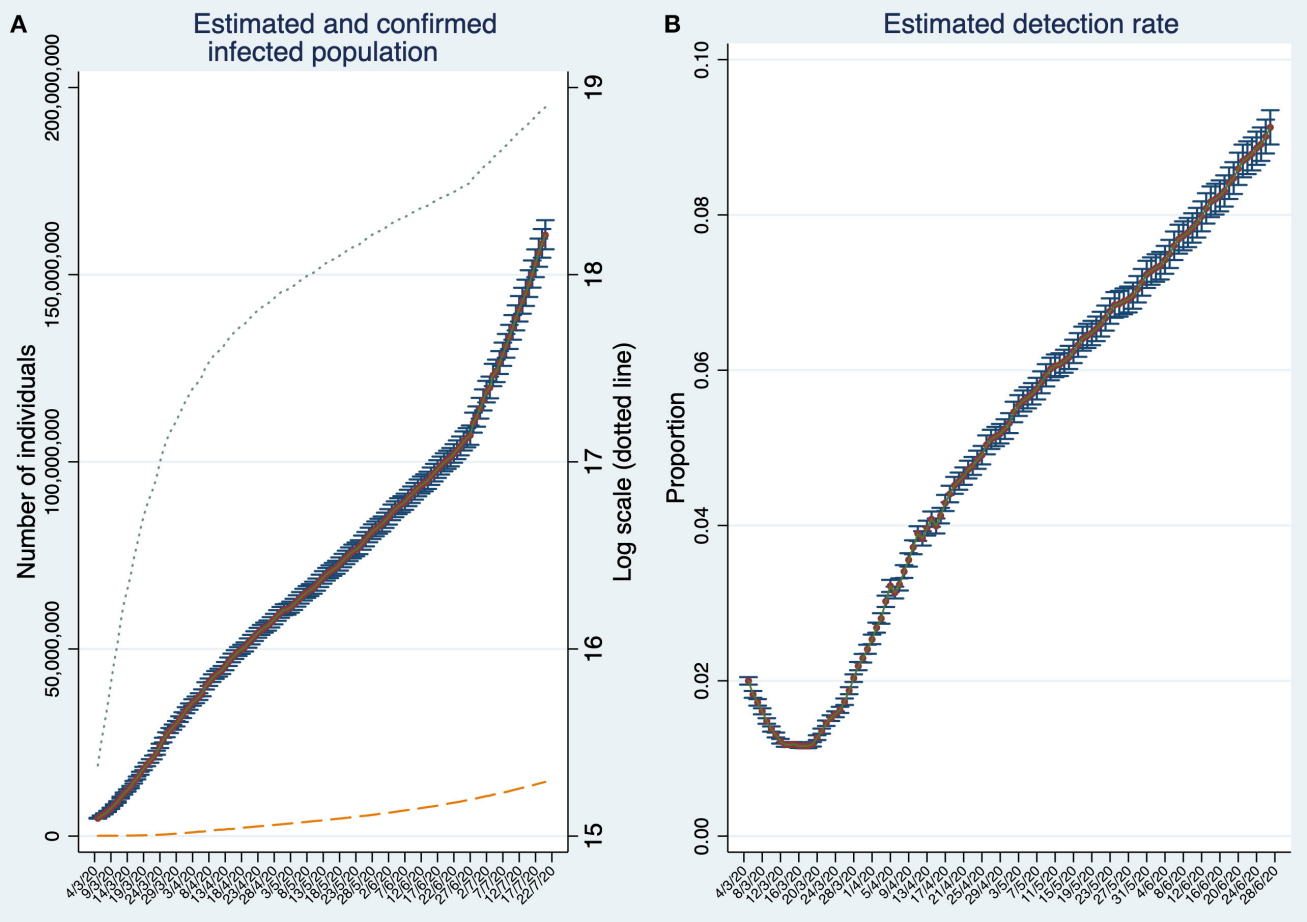
Estimates of the number of SARS-CoV-2 infections and the estimated detection rate in the World. **(A)** Estimated and confirmed infected population. **(B)** Estimated Global detection rate. Note that **(B)** depicts the detection rates until *t*_−23_. Both panels display 95% confidence intervals. Source: Own elaboration.

On July 20, the estimated infected population reaches about 160 million individuals (Figure 2A). This number is about 19 times larger than the reported number of confirmed cases (about 8.6 million represented by the dashed line). Note that the number of infections is estimated based on detection rates calculated 23 days in the past. Thus, for the period *t*_−23_ to *t*, the number of SARS-CoV-2 infected individuals are estimated using the estimation rate as in *t*_−23_. Therefore, the estimation of SARS-CoV-2 infected individuals can be biased if detection rates deteriorate or improve considerably within this time span.

The accuracy of our estimations can be assessed by contrasting them against to those provided by population-based seroepidemiological studies. There are some studies of this type focusing on restricted geographical areas, for instance, in Germany and Switzerland (34, 45). However, to the best of our knowledge, there is only one country level and large scale population-based seroepidemiological study performed in Spain (46). The ENE-COVID study in Spain finds that, on 11 May, 5% of the population would test IgG positive against SARS-CoV-2. It implies that about 2.35 million individuals were infected by SARS-CoV-2. Similarly, in our study we estimated on 11 May an infected population of about 2.25 million individuals. This evidence suggests that our method can be a suitable alternative when population-based seroepidemiological studies are not available, which is frequently the case. Here, it is important to recognize that, from the public health point of view, it is preferable to be vaguely right than precisely wrong. On 11 May, Spain confirmed only 246,504 cases (about 10% of all estimated infections). At that time, it would have been convenient that public health authorities and the public opinion would have the information that, for each confirmed case, there were significantly much more individuals spreading the infection in unpredictable ways.

Back to the global estimates, by comparing the cumulative number of estimated infections with the cumulative number of confirmed (detected) cases, we obtain, at the end of June 2020, a global detection rate of about 9% (Figure 2B). The global detection rate curve shows an U-shape with a minimum at the beginning of the third week of March reaching only 1.1%. The last data suggest that detection rates are steadily increasing. Moreover, the semi-logarithmic plot in Figure 2A suggests that the infection stopped spreading at its maximum pace approximately during the third week of March, but unfortunately, it increased its speed again around the last week of June.

**Figure 3:**
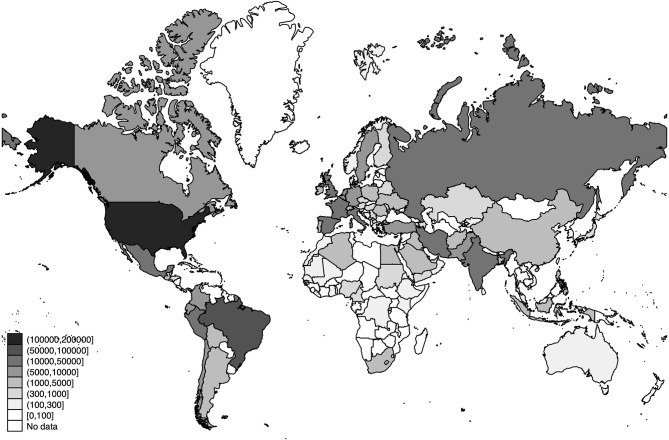
World distribution of deaths as of 20 July 2020. Source: Own Elaboration.

**Figure 4:**
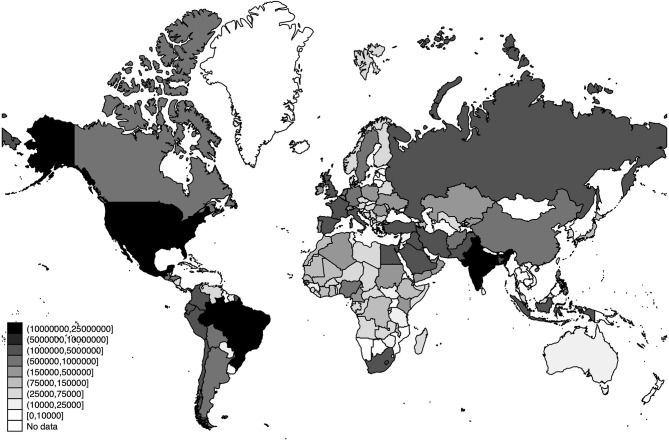
World distribution of the estimated number of SARS-CoV-2 infections as of 20 July 2020. Source: Own Elaboration.

**Figure 5:**
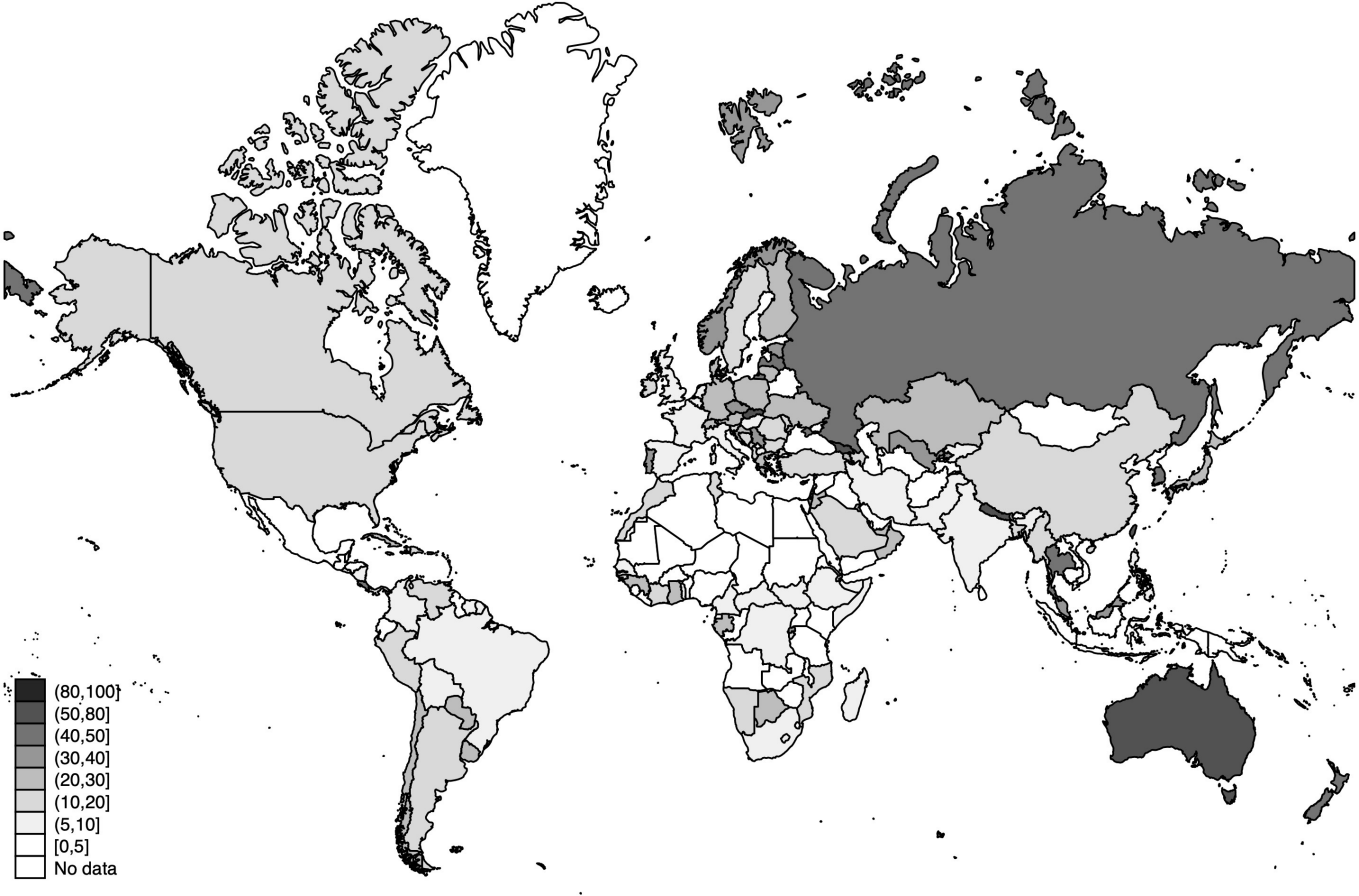
World distribution of the estimated detection rates of SARS-CoV-2 infections as of 20 July 2020 (in percentage). Source: Own Elaboration.

The world distribution of the number of deaths, the estimated number of SARS-CoV-2 infections, and the detection rates of SARS-CoV-2 infections across the world are displayed in Figures 3–5, respectively.

Since the global estimates are no more than an aggregation of the trajectories made by the different countries in the world, we investigate how heterogeneous the detection rates across countries are. Table 1 presents this information in a synchronic way. The rankings compare countries in the same phase of their respective pandemic processes, that is after 15, 30, 45, 60, 75, and 90 days after the confirmation of the first 100 SARS-CoV-2 infections (pandemic outbreak). This approach allows us to perform such an international comparison.

**Table 1A:** Synchronic descriptive statistics (15, 30, and 45 days after the pandemic outbreak).

**Country/Days since the first 100 cases were confirmed**	**Detection rankings**			**Confirmed Cases (in thousands)**			**Estimated Cases (in thousands)**			**Estimated detection rate (Percentage)**			**Number of deaths (Count)**		
	**15**	**30**	**45**	**15**	**30**	**45**	**15**	**30**	**45**	**15**	**30**	**45**	**15**	**30**	**45**
South Korea	1	2	3	6.3	8.8	10.2	15.8	22.5	25.3	39.8	39.1	40.4	42	103	183
Australia	2	1	1	1.8	6.0	6.7	6.7	9.7	10.4	27.3	61.1	64.1	7	45	74
Luxembourg	3	6	7	2.2	3.4	3.8	9.3	11.2	12.3	23.3	30.0	30.9	23	69	90
Thailand	4	3	2	1.4	2.6	2.9	6.0	6.9	7.0	23.0	37.8	41.7	7	41	54
Lithuania	5	5	12	0.8	1.3	1.4	3.4	4.1	5.4	23.0	32.4	26.3	9	36	46
Croatia	6	12	13	1.0	1.8	2.1	4.5	7.4	8.3	22.6	24.4	25.3	7	36	77
Estonia	7	8	8	0.6	1.3	1.6	3.4	4.7	5.5	18.7	27.9	30.1	1	25	50
Norway	8	7	6	1.7	5.5	7.1	10.2	19.2	22.7	17.0	28.7	31.2	7	50	154
Finland	9	24	23	1.0	2.8	4.5	7.2	18.4	24.0	13.3	15.0	18.6	4	48	186
Israel	10	4	5	3.0	10.7	15.4	24.2	33.1	39.0	12.5	32.5	39.5	10	101	199
Czech R.	11	10	11	2.1	5.7	7.4	16.6	22.7	27.2	12.4	25.3	27.1	9	119	218
Japan	12	21	30	0.4	1.0	3.7	3.4	6.8	24.2	12.1	15.4	15.1	6	36	73
Greece	13	15	18	0.9	2.0	2.5	7.8	10.8	12.3	11.4	18.7	20.3	26	90	130
Chile	14	13	24	2.4	7.9	14.9	21.7	38.7	85.5	11.3	20.5	17.4	8	92	216
Austria	15	11	10	3.6	12.3	14.8	33.4	50.4	54.4	10.9	24.4	27.3	16	220	463
Bosnia & H.	16	31	33	0.7	1.3	1.9	6.1	11.9	15.1	10.8	11.0	12.9	24	48	79
Albania	17	18	17	0.4	0.6	0.8	3.6	3.6	3.9	10.4	16.8	21.6	22	26	31
Slovenia	18	25	28	0.6	1.2	1.4	6.3	8.2	8.8	10.1	14.5	16.0	9	50	82
Bulgaria	19	30	31	0.5	0.8	1.6	4.7	7.7	11.0	9.8	11.0	14.5	10	41	72
Puerto Rico	20	26	22	0.8	1.4	2.3	8.1	10.3	11.6	9.8	13.3	19.4	42	84	113
Cuba	21	19	19	0.6	1.4	1.8	7.3	8.4	8.8	8.5	16.3	20.2	16	54	77
Malaysia	22	16	16	1.5	4.0	5.5	18.3	22.5	24.7	8.3	17.6	22.4	14	63	93
Tunisia	23	29	37	0.6	0.9	1.0	7.2	8.1	8.7	8.2	11.1	11.8	22	38	44
Serbia	24	9	4	1.2	5.7	9.4	14.7	20.9	23.2	8.0	27.3	40.3	31	110	189
Portugal	25	14	14	4.3	16.0	23.9	54.1	80.5	94.5	7.9	19.8	25.3	76	470	903
Suriname	26	–	–	0.3	–	–	3.9	–	–	7.8	–	–	8	–	–
Switzerland	27	17	20	4.8	20.2	27.7	75.8	119.4	137.7	6.4	16.9	20.1	43	540	1,134
Moldova	28	35	39	1.0	2.6	4.5	15.4	26.9	39.9	6.3	9.7	11.2	19	73	143
South Africa	29	51	58	1.4	2.6	6.0	22.3	52.4	121.0	6.2	5.0	4.9	5	48	116
Ukraine	30	23	21	1.7	7.6	14.2	28.4	50.6	72.0	5.9	15.1	19.7	52	193	361
Nicaragua	31	39	–	1.1	2.0	–	19.8	24.4	–	5.6	8.3	–	46	64	–
Macedonia	32	33	42	0.5	1.2	1.5	8.7	11.6	14.9	5.6	10.6	10.2	17	54	86
Denmark	33	28	26	1.5	5.1	7.9	27.2	39.7	47.2	5.4	12.8	16.8	24	203	384
El Salvador	34	41	34	0.2	0.7	1.7	4.7	8.7	14.0	5.0	8.0	12.3	8	15	33
Libya	35	53	–	0.4	0.7	–	8.3	16.5	–	4.7	4.2	–	5	18	–
Panama	36	36	36	1.3	4.0	6.7	28.5	43.9	56.6	4.6	9.2	11.9	32	109	192
Poland	37	34	32	1.6	6.7	11.9	35.7	67.3	90.4	4.6	9.9	13.2	18	232	562
Argentina	38	47	51	1.1	2.7	4.7	27.6	44.7	69.6	4.1	5.9	6.7	34	122	237
Bangladesh	39	43	45	2.9	10.9	26.7	73.0	151.7	297.2	4.0	7.2	9.0	101	183	386
Russia	40	27	9	2.3	24.5	106.5	60.6	188.2	370.0	3.9	13.0	28.8	17	198	1,073
Guatemala	41	66	79	0.4	0.9	3.1	10.2	33.4	123.4	3.8	2.7	2.5	11	24	55
China	42	20	25	14.4	70.6	80.3	383.7	455.1	473.8	3.8	15.5	16.9	304	1,771	2,946
Romania	43	38	40	1.5	6.3	11.3	41.4	75.9	104.7	3.5	8.3	10.8	29	306	631
Turkey	44	32	29	15.7	74.2	122.4	471.0	677.4	786.3	3.3	11.0	15.6	277	1,643	3,258
Saudi Arabia	45	44	27	1.2	4.9	20.1	38.6	74.0	124.3	3.2	6.7	16.2	8	65	152
Germany	46	22	15	3.8	57.3	125.1	123.2	374.0	545.7	3.1	15.3	22.9	8	455	2,969
Haiti	47	37	38	0.5	2.5	4.7	17.5	29.0	40.8	3.0	8.6	11.5	21	48	82
Ireland	48	46	41	2.4	9.7	19.6	81.9	159.7	187.4	2.9	6.0	10.5	36	334	1,102
Morocco	49	42	35	1.0	3.0	5.2	34.8	39.6	42.6	2.9	7.7	12.3	70	143	191
South Sudan	50	40	43	0.3	1.3	1.9	12.0	16.4	18.7	2.8	8.0	10.1	6	14	34
Dominican R.	51	49	46	1.6	4.7	8.2	57.9	83.7	98.7	2.7	5.6	8.3	77	226	346
Canada	52	45	48	3.4	20.7	43.9	125.1	340.8	552.5	2.7	6.1	7.9	35	509	2,302
Hungary	53	52	53	0.7	1.9	3.0	25.5	38.6	45.9	2.7	5.0	6.6	32	189	351
Colombia	54	56	55	1.1	3.2	7.0	40.2	79.5	130.0	2.6	4.1	5.4	17	144	314
Niger	55	77	81	0.6	0.8	0.9	26.7	36.6	37.7	2.4	2.1	2.4	19	36	55
Pakistan	56	64	62	1.6	6.0	15.8	71.6	203.6	377.3	2.3	2.9	4.2	18	107	346
U. Arab E.	57	48	44	0.7	5.4	12.5	29.5	91.6	128.4	2.3	5.9	9.7	6	33	105
Sweden	58	60	56	1.6	6.4	14.4	77.9	197.1	287.1	2.1	3.3	5.0	16	373	1,540
Ecuador	59	74	64	2.3	7.9	24.9	118.0	359.2	652.6	2.0	2.2	3.8	79	388	900
Somalia	60	61	61	0.6	1.4	2.0	31.8	43.8	46.9	1.9	3.1	4.2	28	55	78
Bolivia	61	71	68	0.4	1.1	3.1	19.7	46.5	92.4	1.8	2.3	3.4	28	55	142
Burkina Faso	62	76	80	0.4	0.6	0.7	25.5	29.5	30.7	1.6	2.1	2.4	23	41	48
Honduras	63	87	76	0.4	0.7	2.0	24.8	44.4	70.7	1.6	1.5	2.8	25	61	116
Iraq	64	62	73	0.5	1.4	1.8	36.3	44.6	61.2	1.5	3.1	3.0	42	78	88
Sierra Leone	65	63	67	0.3	0.8	1.1	23.0	26.0	30.4	1.5	3.0	3.6	20	45	50
Kenya	66	86	85	0.2	0.4	0.8	16.1	27.4	45.7	1.5	1.5	1.8	11	21	50
Cameroon	67	73	77	0.8	1.8	2.8	57.1	82.5	107.3	1.4	2.2	2.6	12	59	136
D. R. Congo	68	79	71	0.3	0.6	1.4	19.3	31.1	42.0	1.4	1.8	3.3	22	31	61
Algeria	69	72	70	1.3	2.7	4.8	102.5	122.5	147.8	1.3	2.2	3.3	152	384	470
Mauritania	70	59	65	0.7	2.2	4.5	53.4	64.6	120.3	1.3	3.4	3.7	31	95	129
Netherlands	71	54	50	3.0	16.6	32.7	239.8	395.9	482.8	1.2	4.2	6.8	106	1,651	3,684
Iran	72	68	59	9.0	29.4	68.2	733.6	1,167.9	1,440.0	1.2	2.5	4.7	354	2,234	4,232
Mali	73	85	86	0.4	0.7	1.1	30.9	46.3	63.5	1.2	1.5	1.7	21	38	67
Chad	74	80	84	0.5	0.8	0.9	41.2	43.5	43.5	1.2	1.8	2.0	50	65	73
Afghanistan	75	83	74	0.6	1.5	4.7	48.8	96.5	159.4	1.1	1.6	2.9	18	57	122
Peru	76	58	54	1.1	11.5	37.0	106.5	319.1	627.9	1.0	3.6	5.9	30	254	1,051
Sudan	77	84	82	0.8	2.7	5.5	80.1	177.8	234.9	1.0	1.5	2.3	45	111	314
Philippines	78	67	69	1.1	4.6	7.8	118.9	177.0	234.0	0.9	2.6	3.3	68	297	511
Brazil	79	81	83	3.9	22.2	66.5	433.6	1,333.9	3,175.9	0.9	1.7	2.1	114	1,223	4,543
Indonesia	80	75	72	1.3	4.6	9.5	148.1	215.2	307.2	0.9	2.1	3.1	114	399	773
Italy	81	55	52	7.4	63.9	135.6	899.6	1,566.6	2,024.0	0.8	4.1	6.7	366	6,077	17,129
Spain	82	50	47	9.2	94.4	181.5	1,205.5	1,865.5	2,182.4	0.8	5.1	8.3	309	8,189	18,893
India	83	69	66	1.3	11.4	33.1	164.6	456.0	899.4	0.8	2.5	3.7	32	377	1,074
Egypt	84	82	78	0.6	2.2	5.0	77.0	136.9	203.1	0.7	1.6	2.5	36	164	359
Belgium	85	65	57	2.3	18.4	38.5	316.7	634.0	770.7	0.7	2.9	5.0	37	1,283	5,683
Nigeria	86	88	75	0.3	1.5	5.0	54.2	111.0	175.1	0.6	1.4	2.8	10	44	164
France	87	70	60	4.5	40.2	98.1	742.3	1,732.2	2,149.8	0.6	2.3	4.6	91	2,606	14,967
Mexico	88	89	87	1.4	6.3	20.7	251.9	691.8	1,525.6	0.5	0.9	1.4	37	486	1,972
U.K.	89	78	63	3.3	38.2	114.2	907.2	1,904.7	2,945.9	0.4	2.0	3.9	144	3,605	15,464
U.S.	90	57	49	4.7	189.6	639.7	1,547.1	5,216.8	8,058.6	0.3	3.6	7.9	85	4,079	30,985
Yemen	91	90	88	0.3	0.7	1.1	126.8	170.9	242.1	0.2	0.4	0.5	66	160	302

*This ranking is made up of all countries with more than 30 deaths due to COVID-19 40 days after the pandemic outbreak. Countries are ranked by their detection rates 15 days after the pandemic outbreak. Missing values in this table indicate that the country has not reached the requested number of days after its pandemic outbreak. Source: Own elaboration*.

**Table 1B:** Synchronic descriptive statistics (60, 75, and 90 days after the pandemic outbreak).

**Country/Days since the first 100 cases were confirmed**	**Detection rankings**			**Confirmed Cases (in thousands)**			**Estimated Cases (in thousands)**			**Estimated detection rate (Percentage)**			**Number of deaths (Count)**		
	**60**	**75**	**90**	**60**	**75**	**90**	**60**	**75**	**90**	**60**	**75**	**90**	**60**	**75**	**90**
South Korea	6	7	6	10.7	10.8	11.1	26.9	27.9	28.8	39.7	38.7	38.6	236	254	263
Australia	1	1	1	6.9	7.1	7.3	10.8	10.8	11.0	63.8	65.7	65.9	97	101	102
Luxembourg	8	9	10	3.9	4.0	4.1	12.4	12.4	12.4	31.7	32.5	32.9	104	110	110
Thailand	3	4	4	3.0	3.1	3.1	7.3	7.3	7.3	41.4	42.0	42.9	56	57	58
Lithuania	14	16	15	1.6	1.7	1.8	6.1	6.4	6.5	25.8	26.4	27.6	60	71	76
Croatia	16	18	22	2.2	2.2	2.3	8.8	8.8	9.5	25.4	25.4	23.7	95	103	107
Estonia	9	10	9	1.7	1.8	2.0	5.9	5.9	5.9	29.6	31.2	33.4	61	66	69
Norway	7	8	8	7.8	8.3	8.4	23.3	24.0	24.7	33.6	34.3	34.1	208	233	237
Finland	19	19	17	6.0	6.6	7.0	25.6	26.1	26.3	23.3	25.3	26.7	267	308	324
Israel	5	6	5	16.5	16.8	18.4	41.0	42.7	45.9	40.3	39.3	40.0	258	281	299
Czech R.	10	11	12	8.1	9.0	9.8	29.6	30.5	32.1	27.5	29.5	30.4	280	317	328
Japan	13	12	14	11.1	15.4	16.4	42.8	54.5	57.6	26.0	28.2	28.5	186	543	777
Greece	24	25	24	2.7	2.9	3.1	13.4	14.0	14.3	20.3	20.6	21.3	151	172	183
Chile	27	33	27	37.0	90.6	167.4	209.2	608.1	866.7	17.7	14.9	19.3	358	944	3,101
Austria	12	13	16	15.7	16.3	16.8	58.1	58.9	61.0	26.9	27.7	27.5	608	633	672
Bosnia & H.	35	34	36	2.3	2.6	3.3	16.0	17.6	21.7	14.6	14.7	15.1	135	158	168
Albania	20	26	31	1.0	1.2	1.9	4.2	6.4	11.1	23.0	18.9	17.0	31	33	43
Slovenia	31	32	33	1.5	1.5	1.5	9.1	9.2	9.4	16.0	16.0	15.9	102	106	109
Bulgaria	33	35	34	2.2	2.5	3.5	14.1	17.7	22.2	15.8	14.2	15.6	110	144	181
Puerto Rico	11	5	3	3.3	5.3	6.9	12.2	12.8	15.3	27.2	41.7	44.9	129	143	151
Cuba	22	20	19	2.0	2.2	2.3	9.0	9.1	9.3	21.7	24.2	24.9	82	83	85
Malaysia	15	15	11	6.5	7.1	8.3	25.4	26.7	26.7	25.5	26.7	31.1	107	115	117
Tunisia	41	43	42	1.0	1.1	1.2	8.9	9.0	9.1	11.8	12.0	12.7	47	49	50
Serbia	2	2	7	10.6	11.4	12.4	24.3	25.5	34.2	43.7	44.8	36.4	230	244	256
Portugal	17	14	13	27.7	31.0	35.6	109.6	115.5	121.1	25.2	26.9	29.4	1,144	1,342	1,495
Switzerland	23	24	25	29.9	30.5	30.8	145.4	147.8	148.4	20.6	20.7	20.8	1,476	1,613	1,659
Moldova	39	38	35	6.7	9.2	14.0	55.1	73.4	89.8	12.2	12.6	15.5	233	323	464
South Africa	64	60	55	14.4	32.7	73.5	304.5	592.7	1,015.7	4.7	5.5	7.2	261	683	1,568
Ukraine	21	21	21	20.6	27.0	37.2	91.7	119.4	151.1	22.4	22.6	24.6	605	788	1,012
Macedonia	48	51	47	1.9	2.6	4.8	20.9	33.8	46.6	8.9	7.7	10.3	110	147	222
Denmark	25	22	23	10.1	11.2	11.9	50.2	52.5	53.2	20.1	21.4	22.4	514	561	587
El Salvador	46	49	–	2.9	4.6	–	31.1	53.3	–	9.4	8.7	–	53	107	–
Panama	37	37	37	9.4	13.5	21.4	73.6	99.2	151.6	12.8	13.6	14.1	269	336	448
Poland	30	29	26	16.9	22.5	28.2	105.0	125.4	142.1	16.1	17.9	19.8	839	1,028	1,215
Argentina	52	45	40	7.8	17.4	34.1	106.9	161.7	254.4	7.3	10.8	13.4	366	556	878
Bangladesh	38	31	32	57.6	105.5	159.7	460.1	638.5	965.1	12.5	16.5	16.5	781	1,388	1,997
Russia	4	3	2	262.8	396.6	529.0	639.9	896.2	1,146.1	41.1	44.2	46.2	2,418	4,555	6,948
Guatemala	78	76	–	7.1	13.8	–	239.1	417.0	–	3.0	3.3	–	252	547	–
China	29	40	43	81.1	82.4	83.8	480.8	667.4	667.6	16.9	12.3	12.5	3,241	3,316	4,636
Romania	36	36	41	15.8	18.6	21.2	119.2	136.2	158.9	13.2	13.7	13.3	1,002	1,219	1,369
Turkey	28	28	28	148.1	163.9	179.8	853.5	906.0	956.9	17.3	18.1	18.8	4,096	4,540	4,825
Saudi Arabia	26	27	30	44.8	80.2	119.9	227.4	435.6	678.5	19.7	18.4	17.7	273	441	893
Germany	18	17	18	157.6	172.2	180.5	626.6	662.7	680.8	25.2	26.0	26.5	6,115	7,723	8,450
Haiti	40	–	–	6.1	–	–	51.6	–	–	11.8	–	–	110	–	–
Ireland	42	42	44	23.2	24.8	25.2	198.5	204.4	207.7	11.7	12.1	12.2	1,488	1,631	1,703
Morocco	32	30	29	7.1	8.0	9.6	44.7	46.4	52.7	16.0	17.3	18.2	194	208	213
Dominican R.	43	41	39	13.2	18.0	24.6	116.8	148.3	183.3	11.3	12.2	13.4	441	516	635
Canada	45	44	45	67.7	84.7	96.2	700.1	784.8	820.9	9.7	10.8	11.7	4,693	6,424	7,835
Hungary	53	52	53	3.6	3.9	4.1	50.1	52.4	53.9	7.1	7.5	7.6	467	534	568
Colombia	54	54	60	14.9	29.4	53.1	233.8	429.7	809.2	6.4	6.8	6.6	562	939	1,726
Niger	81	80	–	1.0	1.0	–	38.9	39.5	–	2.5	2.6	–	65	67	–
Pakistan	60	62	52	37.2	66.5	139.2	686.1	1,230.3	1,658.4	5.4	5.4	8.4	803	1,395	2,632
U. Arab E.	34	23	20	21.8	33.9	42.3	145.2	159.4	171.5	15.0	21.3	24.7	210	262	289
Sweden	55	53	50	22.7	30.8	40.8	365.8	417.2	457.6	6.2	7.4	8.9	2,769	3,743	4,542
Ecuador	70	71	71	31.5	38.6	46.8	763.3	890.2	1,027.2	4.1	4.3	4.6	2,594	3,334	3,896
Somalia	57	59	–	2.6	2.9	–	48.0	51.8	–	5.5	5.7	–	88	90	–
Bolivia	62	61	65	8.4	16.9	30.7	160.1	310.3	534.0	5.2	5.5	5.7	293	559	970
Burkina Faso	80	78	74	0.8	0.9	0.9	30.7	30.7	30.9	2.7	2.9	2.9	52	53	53
Honduras	67	74	68	4.4	7.4	15.4	103.6	182.8	316.5	4.2	4.0	4.9	188	290	426
Iraq	82	82	76	3.0	5.5	17.8	124.6	442.8	1,081.1	2.4	1.2	1.6	115	179	496
Sierra Leone	68	–	–	1.4	–	–	33.3	–	–	4.2	–	–	59	–	–
Kenya	79	73	72	2.0	3.7	6.4	68.7	90.8	147.6	2.9	4.1	4.3	64	104	148
Cameroon	73	64	64	5.4	9.2	12.6	158.6	173.4	215.9	3.4	5.3	5.8	177	273	313
D. R. Congo	65	63	63	3.0	4.8	6.9	66.6	89.8	117.8	4.5	5.3	5.9	69	106	167
Algeria	66	69	69	7.5	9.8	11.5	176.2	209.3	237.2	4.3	4.7	4.9	568	681	825
Netherlands	51	50	51	40.8	44.2	46.7	514.4	529.6	534.8	7.9	8.4	8.7	5,082	5,715	5,977
Iran	59	57	61	89.3	107.6	137.7	1,638.3	1,843.7	2,132.3	5.5	5.8	6.5	5,650	6,640	7,451
Mali	83	79	–	1.6	2.0	–	69.4	74.8	–	2.3	2.7	–	94	112	–
Chad	84	–	–	0.9	–	–	44.4	–	–	2.0	–	–	74	–	–
Afghanistan	71	67	66	11.8	22.1	30.2	296.0	443.9	591.6	4.0	5.0	5.1	220	405	675
Peru	49	46	46	84.5	155.7	229.7	1,017.4	1,497.7	2,038.9	8.3	10.4	11.3	2,393	4,371	6,688
Sudan	75	77	–	8.3	9.7	–	266.9	311.7	–	3.1	3.1	–	506	604	–
Philippines	69	68	58	11.4	15.0	24.2	273.5	314.0	358.5	4.2	4.8	6.7	751	904	1,036
Brazil	76	66	54	177.6	411.8	802.8	5,729.2	8,243.9	10,800.0	3.1	5.0	7.4	12,400	25,598	40,919
Indonesia	72	72	70	15.4	24.5	36.4	425.5	583.9	762.3	3.6	4.2	4.8	1,028	1,496	2,048
Italy	50	48	49	187.3	215.9	228.7	2,287.7	2,412.9	2,485.6	8.2	8.9	9.2	25,085	29,958	32,616
Spain	47	47	48	215.2	230.7	239.4	2,381.4	2,247.5	2,345.9	9.0	10.3	10.2	24,824	27,563	27,127
India	63	65	59	82.0	173.8	320.9	1,675.2	3,312.0	4,874.1	4.9	5.2	6.6	2,649	4,971	9,195
Egypt	77	75	73	10.4	20.8	41.3	340.3	614.6	975.5	3.1	3.4	4.2	556	845	1,422
Belgium	56	55	57	50.3	55.8	58.7	823.7	847.7	857.3	6.1	6.6	6.8	7,924	9,108	9,522
Nigeria	74	70	67	8.9	15.2	24.1	266.1	339.2	486.8	3.4	4.5	4.9	259	399	558
France	61	58	62	126.8	140.7	149.1	2,350.2	2,424.9	2,468.3	5.4	5.8	6.0	23,660	27,074	28,662
Mexico	85	81	75	47.1	90.7	150.3	2,899.6	4,823.5	6,766.9	1.6	1.9	2.2	5,045	9,930	17,580
U.K.	58	56	56	186.6	246.4	278.0	3,403.7	3,778.5	3,971.6	5.5	6.5	7.0	28,446	34,796	39,369
U.S.	44	39	38	1,069.8	1,443.4	1,770.4	10,137.1	11,539.1	12,571.5	10.6	12.5	14.1	63,006	87,568	103,781

*This ranking is made up of all countries with more than 30 deaths due to COVID-19 40 days after the pandemic outbreak. Countries are ranked by their detection rates 15 days after the pandemic outbreak. Missing values in this table indicate that the country has not reached the requested number of days after its pandemic outbreak. Source: Own elaboration*.

At a first sight, it is noteworthy the fact that each of the first 24 countries ranked on the top by the initial detection rate (15 days after the beginning of the pandemic outbreak) does not accumulate more than 500 deaths 45 days after initiating their pandemic processes. Thus, it seems to exist a strong correlation between detection rates and the cumulative number of deaths for a given stage of the pandemic process. Countries with high counts of deaths ranked very badly in their initial detection rates. For example, the US, Spain, Italy, UK, France, and Belgium ranked in place 90, 82, 81, 89, 87, and 85, out of 91 countries listed in the ranking.

A second conclusion is that the relative improvement of detection rates over time, that is, 30, 45, 60, 75, and 90 days after the beginning of the pandemic processes, does not alter the fact that those countries are still ranked the worst in terms of deaths. That is, improving detection over time has declining returns to scale when comes to save lives.

The depicted relationship between detection rates and the cumulative number of deaths remains almost unchanged when using non-synchronic data as of 20 May in Table 2. This table mixes information of countries at different stages from their pandemic processes. So, it must be interpreted with caution. Although efforts to increase detection have been significative in the above-mentioned countries, none of them is still ranked on the top part of the ranking with 91 countries for which we have full data (US in ranking 36, Spain 45, Italy 46, Belgium 55, UK 56, and France in place 58). Similarly, in this non-synchronic ranking, with the exception of Russia, none of the first 10 countries accumulated more than 500 deaths on May 20.

**Table 2:** Non-synchronic descriptive statistics as of 20 May.

**Country**	**Detection rate ranking**	**Confirmed Cases**	**Estimated Cases**	**Number of Deaths**	**Detection rate (Percentage)**	**Country**	**Detection rate ranking**	**Confirmed Cases**	**Estimated Cases**	**Number of Deaths**	**Detection rate (Percentage)**
Australia	1	7,068	10,803	99	65.4	Macedonia	47	1,839	20,629	106	8.9
Serbia	2	10,733	24,472	234	43.9	Bangladesh	48	25,121	284,758	370	8.8
Russia	3	299,941	713,396	2,837	42.0	Peru	49	99,483	1,137,309	2,914	8.7
Thailand	4	3,034	7,293	56	41.6	Netherlands	50	44,249	529,581	5,715	8.4
Israel	5	16,650	42,239	277	39.4	Argentina	51	8,796	114,004	393	7.7
South Korea	6	11,110	28,757	263	38.6	Sweden	52	30,799	417,234	3,743	7.4
Norway	7	8,257	24,041	233	34.3	Hungary	53	3,598	50,265	470	7.2
Luxembourg	8	3,958	12,368	109	32.0	Colombia	54	16,935	256,149	613	6.6
Estonia	9	1,791	5,891	64	30.4	Belgium	55	55,791	847,676	9,108	6.6
Czech R.	10	8,647	30,105	302	28.7	U.K.	56	248,818	3,792,371	35,341	6.6
Japan	11	16,385	57,455	771	28.5	Iran	57	124,603	1,992,740	7,119	6.3
Austria	12	16,257	58,599	632	27.7	France	58	143,427	2,444,441	28,022	5.9
Malaysia	13	6,978	26,056	114	26.8	Pakistan	59	45,898	844,102	985	5.4
Germany	14	176,007	671,716	8,090	26.2	India	60	106,750	2,054,585	3,303	5.2
Portugal	15	29,432	113,959	1,247	25.8	South Africa	61	17,200	363,475	312	4.7
Lithuania	16	1,562	6,060	60	25.8	Philippines	62	12,942	287,923	837	4.5
Croatia	17	2,232	8,763	96	25.5	Libya	63	68	1,544	3	4.4
Finland	18	6,399	26,036	301	24.6	Ecuador	64	34,151	784,137	2,839	4.4
Puerto Rico	19	2,805	11,951	124	23.5	Algeria	65	7,377	173,847	561	4.2
Albania	20	949	4,088	31	23.2	Brazil	66	271,628	6,890,826	17,971	3.9
Ukraine	21	18,876	86,905	548	21.7	Bolivia	67	4,481	115,342	189	3.9
Denmark	22	11,044	52,134	551	21.2	Indonesia	68	18,496	480,800	1,221	3.8
Cuba	23	1,887	8,926	79	21.1	D. R. Congo	69	1,731	47,886	61	3.6
Greece	24	2,840	13,671	165	20.8	Somalia	70	1,502	44,338	59	3.4
Switzerland	25	30,535	147,794	1,613	20.7	South Sudan	71	285	8,421	6	3.4
Saudi Arabia	26	59,854	303,501	329	19.7	Egypt	72	13,484	401,828	659	3.4
Turkey	27	151,615	862,911	4,199	17.6	Honduras	73	2,955	88,824	147	3.3
Poland	28	19,268	114,170	948	16.9	Afghanistan	74	7,653	230,911	178	3.3
U. Arab E.	29	25,063	150,496	227	16.7	Nigeria	75	6,401	203,510	192	3.1
Bulgaria	30	2,292	14,227	116	16.1	Cameroon	76	3,529	113,118	140	3.1
Slovenia	31	1,467	9,206	104	15.9	Haiti	77	596	19,339	22	3.1
Morocco	32	7,023	44,481	193	15.8	Burkina Faso	78	806	30,682	52	2.6
Bosnia & H.	33	2,319	15,813	133	14.7	Suriname	79	11	429	1	2.6
Chile	34	49,579	359,514	509	13.8	Niger	80	914	37,746	55	2.4
Romania	35	17,191	125,640	1,126	13.7	Sierra Leone	81	534	24,508	33	2.2
U.S.	36	1,528,568	11,884,244	91,921	12.9	Guatemala	82	2,133	103,309	43	2.1
Panama	37	9,867	77,349	281	12.8	Kenya	83	963	49,412	50	1.9
China	38	84,065	667,702	4,638	12.6	Iraq	84	3,611	199,779	131	1.8
Moldova	39	6,340	51,912	221	12.2	Mexico	85	54,346	3,289,790	5,666	1.7
Ireland	40	24,251	203,128	1,561	11.9	Mali	86	901	57,558	53	1.6
Tunisia	41	1,044	8,865	47	11.8	Sudan	87	2,591	169,575	105	1.5
El Salvador	42	1,498	12,905	31	11.6	Chad	88	545	42,352	56	1.3
Dominican R.	43	13,223	116,836	441	11.3	Mauritania	89	81	27,361	4	0.3
Canada	44	79,101	763,889	5,912	10.4	Yemen	90	167	67,359	28	0.2
Spain	45	232,555	2,247,533	27,888	10.3	Nicaragua	91	25	14,739	8	0.2
Italy	46	226,699	2,472,703	32,169	9.2						

*Countries are ranked by the detection rates of SARS-CoV-2 infections as of 20 May. Source: Own elaboration*.

In Table 3, we present the non-synchronic ranking as of 22 June. The US is in place 35, Spain 49, Italy 53, Belgium 63, UK 61, and France 67. It is noteworthy that, except for Russia, none of the first 16 countries in this ranking have accumulated more than 2,000 fatalities on 22 June. More importantly and despite the incredible efforts to increase the tests amongst the more developed countries, none of them were able to detect more than 16% of the estimated infections (the US detected 15.7% on 22 June). It implies that testing efforts need to be deployed at the first stages of the pandemic process due to its cumulative nature. Tables 2, 3 show that moving over time from relatively low to relatively high cumulative detection rates is unlikely and probably very expensive. This is due to the over proportional efforts needed to expand testing relative to the exponentially growing infections at the early stages of the pandemic. Consequently, from the public health point of view, it is much more advantageous, technically, and economically feasible, to implement mass testing from the very beginning of the pandemic process. To achieve this goal, health authorities and governments would require understanding the linkages between the cumulative detection rates and the minimization of the pandemic related fatalities and economic damage.

**Table 3:** Non-synchronic descriptive statistics as of 22 June.

**Country**	**Detection rate ranking**	**Confirmed Cases**	**Estimated Cases**	**Number of Deaths**	**Detection rate (Percentage)**	**Country**	**Detection rate ranking**	**Confirmed Cases**	**Estimated Cases**	**Number of Deaths**	**Detection rate (Percentage)**
Australia	1	7,461	11,478	102	65.0	Sweden	47	56,043	479,232	5,053	11.7
Russia	2	584,680	1,271,052	8,111	46.0	Peru	48	254,936	2,272,406	8,045	11.2
Puerto Rico	3	6,525	14,521	149	44.9	Spain	49	246,504	2,357,978	28,324	10.5
Thailand	4	3,148	7,301	58	43.1	Macedonia	50	5,106	49,133	238	10.4
South Korea	5	12,438	30,176	280	41.2	South Sudan	51	1,882	18,688	34	10.1
Israel	6	20,778	51,835	306	40.1	Pakistan	52	181,088	1,908,427	3,590	9.5
Norway	7	8,708	25,098	244	34.7	Italy	53	238,499	2,533,481	34,634	9.4
Estonia	8	1,981	5,896	69	33.6	Netherlands	54	49,593	538,868	6,090	9.2
Serbia	9	12,894	38,735	261	33.3	El Salvador	55	4,626	53,327	107	8.7
Luxembourg	10	4,120	12,508	110	32.9	Brazil	56	1,085,038	12,484,118	50,617	8.4
Czech R.	11	10,498	32,666	336	32.1	Nicaragua	57	2,014	24,386	64	8.3
Malaysia	12	8,572	27,040	121	31.7	South Africa	58	97,302	1,269,375	1,930	7.7
Portugal	13	39,133	127,864	1,530	30.6	Hungary	59	4,102	54,281	572	7.6
Japan	14	17,916	61,665	953	29.1	Suriname	60	314	4,170	8	7.5
Austria	15	17,285	61,817	690	28.0	U.K.	61	304,331	4,151,851	42,632	7.3
Lithuania	16	1,798	6,497	76	27.7	India	62	425,282	6,033,057	13,699	7.0
Germany	17	190,359	699,154	8,885	27.2	Belgium	63	60,550	861,976	9,696	7.0
Finland	18	7,143	26,402	326	27.1	Philippines	64	30,052	438,038	1,169	6.9
U. Arab E.	19	44,925	178,155	302	25.2	Iran	65	204,952	3,050,048	9,623	6.7
Cuba	20	2,312	9,281	85	24.9	Colombia	66	68,652	1,030,695	2,237	6.7
Ukraine	21	36,560	148,376	1,002	24.6	France	67	160,377	2,515,344	29,640	6.4
Chile	22	242,355	991,336	4,479	24.4	D. R. Congo	68	5,826	98,916	130	5.9
Croatia	23	2,317	9,957	107	23.3	Cameroon	69	11,610	199,080	301	5.8
Denmark	24	12,391	53,638	600	23.1	Bolivia	70	24,388	424,522	773	5.7
Greece	25	3,266	14,533	190	22.5	Somalia	71	2,779	49,186	90	5.7
Poland	26	31,931	150,582	1,356	21.2	Afghanistan	72	28,833	565,246	581	5.1
Switzerland	27	31,209	148,912	1,680	21.0	Indonesia	73	45,891	905,257	2,465	5.1
Saudi Arabia	28	157,612	823,639	1,267	19.1	Nigeria	74	20,244	409,327	518	4.9
Turkey	29	187,685	984,358	4,950	19.1	Honduras	75	12,769	263,032	363	4.9
Morocco	30	9,977	55,386	214	18.0	Algeria	76	11,771	242,645	845	4.9
Albania	31	1,927	11,300	44	17.1	Egypt	77	55,233	1,182,338	2,193	4.7
Bulgaria	32	3,905	23,586	199	16.6	Ecuador	78	50,640	1,084,641	4,223	4.7
Bangladesh	33	112,306	678,767	1,464	16.5	Kenya	79	4,738	109,829	123	4.3
Slovenia	34	1,520	9,410	109	16.2	Libya	80	544	12,846	10	4.2
U.S.	35	2,280,912	14,248,772	119,975	15.7	Sierra Leone	81	1,327	31,692	55	4.2
Moldova	36	14,200	93,470	473	15.2	Mauritania	82	2,813	75,687	108	3.7
Bosnia & H.	37	3,354	22,225	169	15.1	Guatemala	83	13,145	398,078	531	3.3
Panama	38	26,030	180,819	501	14.4	Sudan	84	8,580	276,728	521	3.1
Argentina	39	42,772	303,341	1,011	14.1	Burkina Faso	85	903	30,773	53	2.9
Romania	40	24,045	177,775	1,512	13.5	Mali	86	1,961	73,306	111	2.7
Dominican R.	41	26,677	197,251	662	13.5	Niger	87	1,036	39,912	67	2.6
Tunisia	42	1,157	9,144	50	12.7	Mexico	88	180,545	7,666,945	21,825	2.4
China	43	84,572	668,564	4,639	12.6	Chad	89	858	43,988	74	2.0
Ireland	44	25,379	208,366	1,715	12.2	Iraq	90	30,868	1,582,972	1,100	2.0
Canada	45	101,326	845,149	8,430	12.0	Yemen	91	941	203,732	256	0.5
Haiti	46	5,211	44,065	88	11.8						

*Countries are ranked by the detection rates of SARS-CoV-2 infections as of 22 June. Source: Own elaboration*.

### Unconditional Analysis

**Figure 6:**
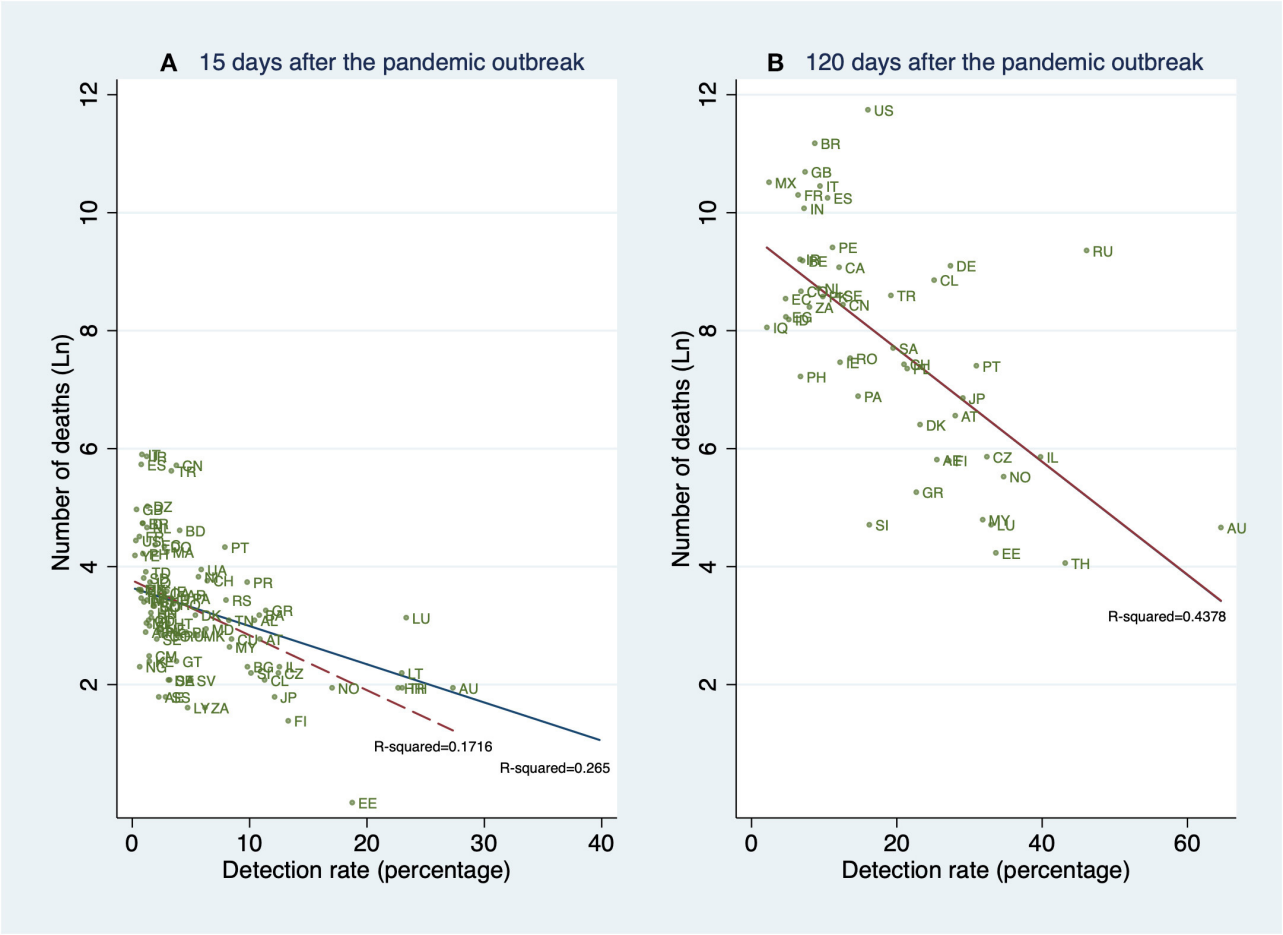
Linear prediction for the natural logarithm of the cumulative number of deaths from a linear regression of ln(*deaths*_*i*_) on the detection rates (*DR*_*i*_) 15 and 120 days after the pandemic outbreak (PO). **(A)** Detection rates and deaths 15 days after the PO. **(B)** Detection rates and deaths 120 days after the PO. **(A)** contains all 91 countries (in Table 1A). **(B)** contains all 61 countries (in Table 1A) whose pandemic processes have more than 120 days since the PO. The dashed fitted line excludes South Korea (KR). Source: Own elaboration.

In this analysis, we show the unconditional relationship between detection rates and deaths. The fitted lines in Figure 6 are obtained after regressing the natural logarithm of the cumulative number of deaths in the country *i* on their estimated cumulative detection rates (*DR*_*i*_). The results strongly suggest a negative relationship between detection rates and the cumulative number of deaths. This strong negative slope is in concordance with the hypothesis that, by detecting a higher proportion of the SARS-CoV-2 infected population, many lives can be saved, in particular, the lives of the elderly and those individuals with preexisting conditions.

The strong association between the number of deaths and the estimated cumulative detection rates remains significant 15, and 120 days after the PO. These associations are shown in Figures 6A,B, respectively.

**Figure 7:**
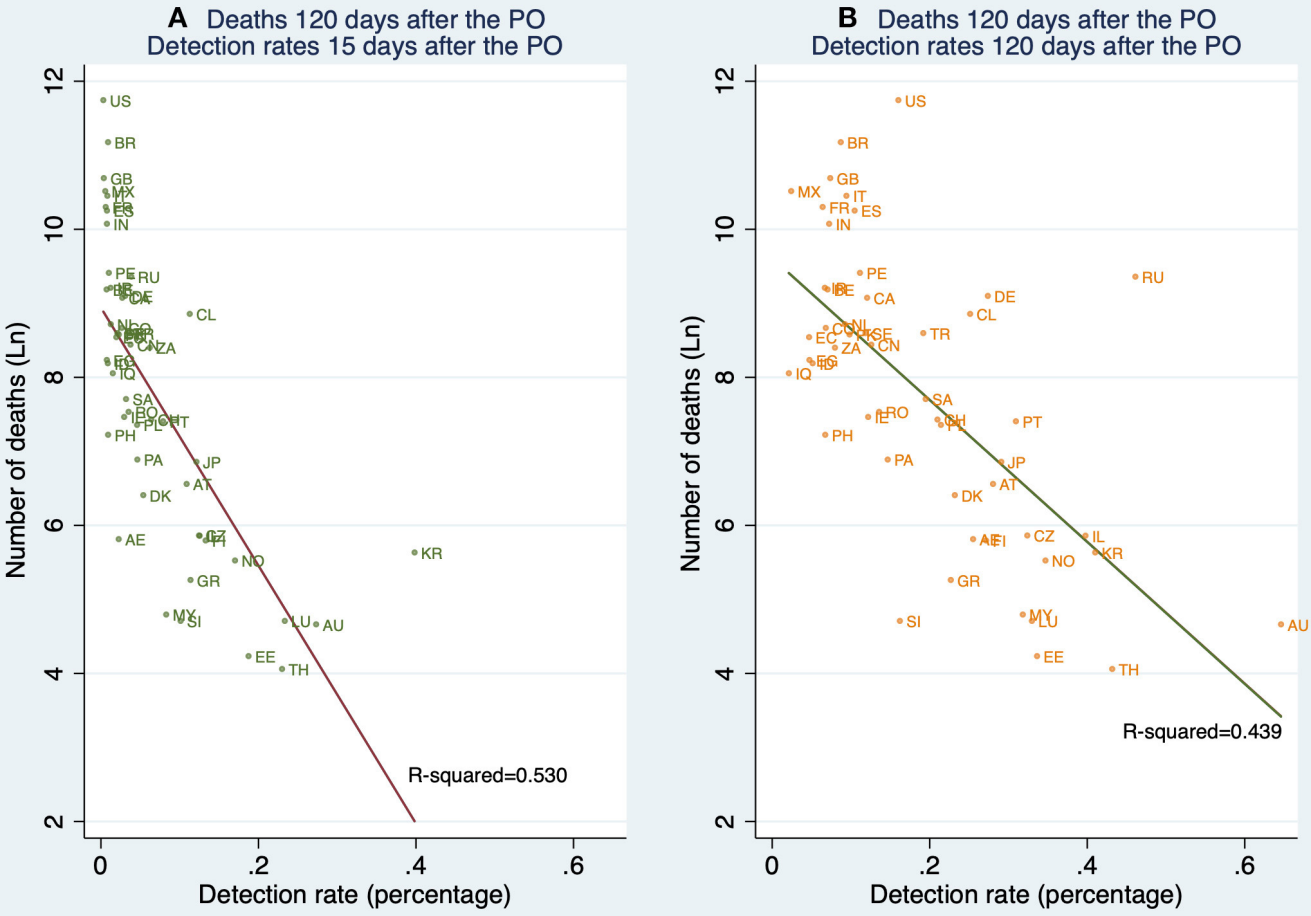
Linear prediction for the natural logarithm of the cumulative number of deaths 120 days after the pandemic outbreak (PO) from a linear regression of ln(*deaths*_*i*_) on the detection rates (*DR*_*i*_) 15 and 120 days after the PO. **(A)** Deaths reported 120 days after the PO, and detection rates estimated 15 days after the PO. **(B)** Deaths reported 120 days after the PO, and detection rates 120 days after the PO. Note: This figure contains all 61 countries (in Table 1A) whose pandemic processes have more than 120 days since the pandemic outbreak. Source: Own elaboration.

Figure 7 shows the relationship between detection rates (15 and 120 days after the PO) and deaths 120 days after the PO. This descriptive result is of interest since it suggests that, unconditionally, early detection is associated with death outcomes 120 days after the PO to a greater extent than the contemporary detection rates, that is, 120 days after the PO.

Although this information suggests the existence of a strong relationship between detection rates and the cumulative number of deaths, this slope may be confounded by the variables mentioned before. Thus, in the next section, we show the results of our conditional analysis as described earlier.

### Multivariate Regression Analysis

Our results in Table 4 show that higher detection rates are associated with a reduction in the number of deaths after controlling for demography (age-structure of the population and population size), economic performance (GDP per capita), and the relative resources that the economies devote to their health systems. Over time, the cross-sectional regressions increase in explanatory power, from a R-squared of 0.71 in model 2 to 0.95 in model 8.

**Table 4:** Synchronic multiple linear regression of the natural logarithm of the cumulative number of deaths on the estimated detections rates.

**Dependent Variable: Ln(deaths)/Explanatory Variables**	**Days since the first 100 cases were confirmed**							
	**15**		**60**			**120**		
	**Model (1)**	**Model (2)**	**Model (3)**	**Model (4)**	**Model (5)**	**Model (6)**	**Model (7)**	**Model (8)**
Estimated detection rate	−0.193^***^	−0.225^***^	−0.120^***^	−0.118^***^	–	−0.100^***^	−0.0976^***^	–
	(0.0358)	(0.0269)	(0.0368)	(0.0435)	–	(0.0220)	(0.0217)	–
Estimated detection rate (Squared)	0.00410^***^	0.00497^***^	0.00135	0.00132	–	0.000948^*^	0.000931^*^	–
	(0.00127)	(0.000698)	(0.000919)	(0.00107)	–	(0.000501)	(0.000484)	–
Estimated detection rate 15 days after PO	–	–	–	–	−22.30^***^	–	–	−16.78^***^
	–	–	–	–	(6.266)	–	–	(5.479)
Estimated detection rate 15 days after PO (squared)	–	–	–	–	47.64^*^	–	–	35.63
	–	–	–	–	(28.54)	–	–	(24.01)
Infection fatality rate	0.960^***^	0.922^***^	1.586^***^	1.512^***^	1.396^***^	1.525^***^	1.506^***^	1.439^***^
	(0.333)	(0.328)	(0.267)	(0.270)	(0.370)	(0.172)	(0.179)	(0.329)
Population size (Ln)	−0.150^**^	−0.146^**^	−0.0285	−0.0179	0.0105	0.0699^**^	0.0649^*^	0.0267
	(0.0656)	(0.0688)	(0.0856)	(0.0780)	(0.0787)	(0.0345)	(0.0356)	(0.0518)
Confirmed cases (Ln)	0.860^***^	0.773^***^	0.943^***^	0.910^***^	0.705^***^	0.931^***^	0.929^***^	0.849^***^
	(0.0995)	(0.108)	(0.0696)	(0.0640)	(0.0796)	(0.0324)	(0.0334)	(0.0639)
GDP per capita (Ln)	−0.446^***^	−0.417^***^	0.0399	0.0570	0.0742	0.181^***^	0.168^**^	−0.0194
	(0.108)	(0.103)	(0.0842)	(0.0913)	(0.138)	(0.0600)	(0.0642)	(0.154)
Health spending as % of GDP	−0.0570^*^	−0.0552	−0.0147	−0.0270	−0.000955	0.00186	−0.0115	0.00210
	(0.0330)	(0.0354)	(0.0231)	(0.0260)	(0.0277)	(0.0143)	(0.0163)	(0.0312)
BCG group 2	–	−0.441	–	0.175	0.124	–	0.185^*^	0.196
	–	(0.323)	–	(0.161)	(0.206)	–	(0.101)	(0.233)
BCG group 3	–	−0.396	–	0.0449	−0.0185	–	0.185	0.197
	–	(0.284)	–	(0.235)	(0.305)	–	(0.165)	(0.332)
BCG group 4	–	−0.704^***^	–	−0.193	−0.205	–	−0.0324	−0.131
	–	(0.220)	–	(0.184)	(0.209)	–	(0.102)	(0.213)
BCG group 5	–	−0.411^*^	–	−0.172	−0.175	–	0.0200	0.0653
	–	(0.210)	–	(0.152)	(0.176)	–	(0.0856)	(0.178)
Constant	4.530^***^	5.355^***^	−2.365	−2.204	−1.313	−5.437^***^	−5.174^***^	−2.425^*^
	(1.391)	(1.434)	(1.431)	(1.442)	(1.629)	(0.679)	(0.776)	(1.362)
Observations	87	87	84	84	84	74	74	74
R-squared	0.672	0.708	0.950	0.954	0.934	0.984	0.985	0.954
R-squared adjusted	0.643	0.666	0.945	0.947	0.924	0.983	0.983	0.946
F-test	26	21.86	342.3	274.9	110.5	594.5	404.1	137.8

Standard errors in parentheses. Significance levels:

*** p < 0.01,

** p < 0.05,

* *p < 0.1. Source: Own elaboration*.

**Figure 8:**
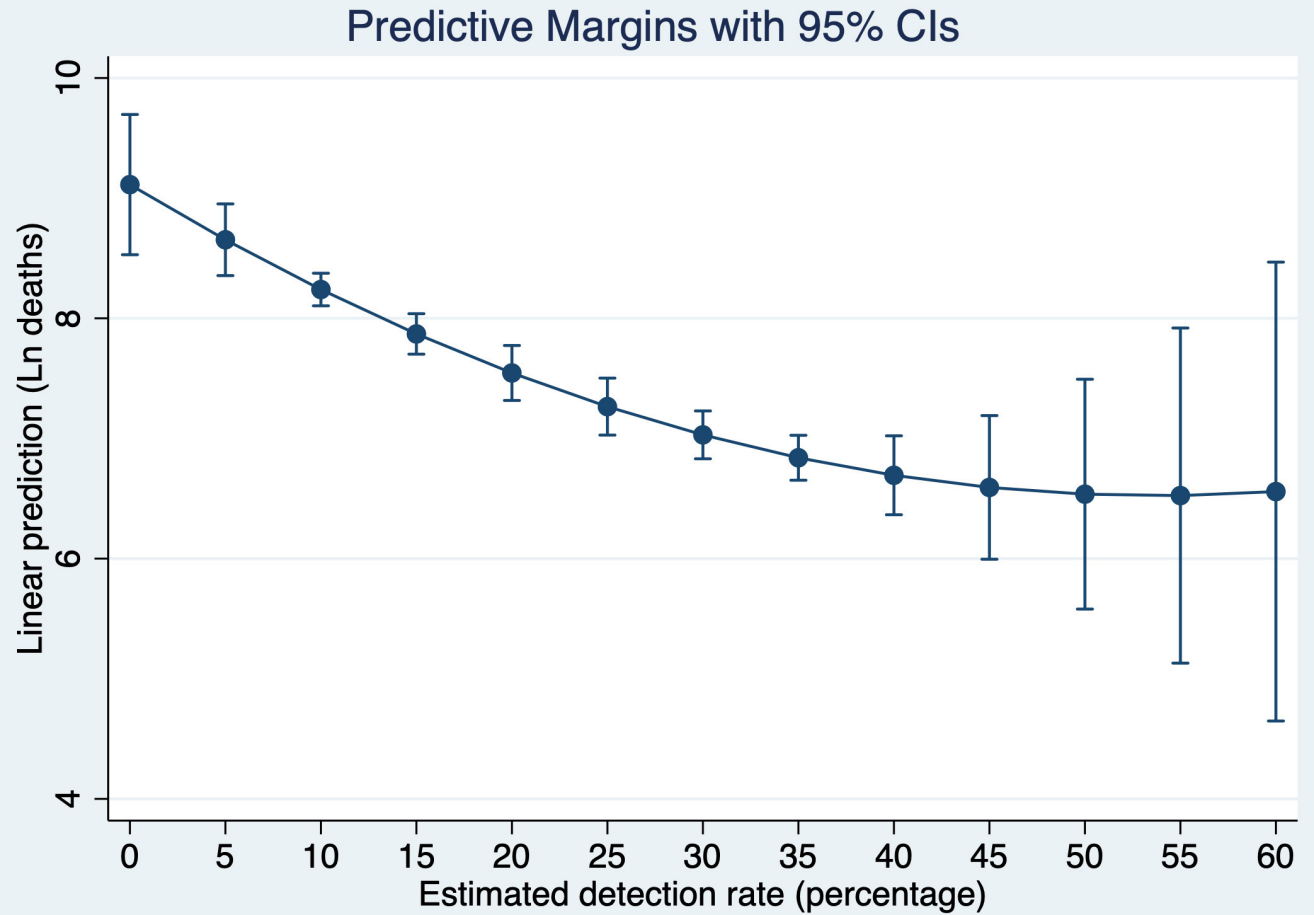
Predictive Margins of the cumulative number of deaths at different detection rates of SARS-CoV-2 infections after 120 days of the pandemic outbreak. Source: Own elaboration.

Based on these results, Figure 8 shows a strong conditional gradient between detection rates and the cumulative number of deaths. For instance, for a hypothetical country with average and constant endowments, the cost in terms of deaths of detecting 5% vs. 35% is about 1.81 natural logarithm points which corresponds to *exp*^1.81^ = 6.13. That is, the average country detecting 5% is associated with a number of deaths about 6.1 times higher when compared with the same country detecting 35% of all SARS-CoV-2 infections.

To put this result in perspective, let us simulate what would be the number of deaths in the U.S., if instead of detecting 16.02% 120 days after the pandemic outbreak, the country would have detected with the same intensity as South Korea (41.01%). Evaluating the number of deaths at the endowments of the U.S, the country would have fewer deaths by 1.14 natural logarithm points. It means that the current U.S deaths are now 3.13 times higher than they would be if the country would have tested with similar intensity as South Korea. Since the number of deaths 120 days after the pandemic outbreak reached 126,140, detecting at the rate of South Korea would have saved about 85,794 lives in the U.S. at that time.

Finally, looking at the regression coefficients in Table 3, it is noteworthy the fact that during the pandemic outbreak, a 1% higher detection rate is associated with more lives saved than a 1% increase in the health expenditure over the GDP. Our results also suggest that the number of deaths, rather than depending on the relative solvency of the health system, could depend in a greater extent on the size and opportunity of the testing efforts.

The conclusion is the more tests the better. Although in this study we employed an economics inspired approach to figure out the importance of testing, our findings are also endorsed by recent medical literature on coronavirus as well as by another economics inspired models providing support to a causal relationship between detection and saving lives (47–50).

### Robustness of the Results

Robust regressions provide estimates that are close to the ones reported in Table 4. Consequently, it is unlikely that the results reported in this study are outlier driven. Additionally, results are robust to heteroscedasticity of unknown form for small samples. Nevertheless, results should be interpreted with caution. The few observations available for the regressions and lack of data does not allow to rule out the possibility that there are omitted variables that have the potential to bias the results.

It is important to keep in mind that results can be biased if omitted variable problem exists. That is, there are variables that are correlated with the explained outcome but at the same time they are also correlated with the explanatory variables of interest. For instance, one can think in countries implementing lockdowns because lower detection rates (Argentina), or relaxed social distancing rules because higher detection rates (Australia). Nevertheless, these non-observed variables yield to an underestimation of the true association between detection rates and the cumulative number of deaths. Thus, detection matters.

## Discussion

In this study, we have proposed a method to estimate the number of SARS-CoV-2 infections for the globe and also for all 91 major countries covering more than 86% of the world population. On June 22, we find that, worldwide, about 160 million individuals have been infected by SARS-CoV-2. Moreover, only about 1 out of 11 these infections have been detected. We find that detection rates are very unequally distributed across the globe and that they also increased over time from about 1% during the second and third weeks of March to about 9% on June 22. In an information context in which population-based seroepidemiological studies are not available, this study shows a parsimonious alternative to provide estimates of the number of SARS-CoV-2 infected individuals. By comparing our estimates with those provided by the ENE-COVID study in Spain, we confirm the utility of our approach keeping in mind that from the public health point of view, it is preferable to be vaguely right than precisely wrong.

In order to provide reliable estimates of the number of SARS-CoV-2 infections and of the cumulative detection rates, it is necessary that governments provide real-time information about the number of COVID-19 deaths. This study supports the view that an accurate communication of the fatality cases can have consequences on the development of the pandemic itself. Thus, it is also a call for allowing international comparison following WHO international norms and standards for medical certificates of COVID-19 cause of death and International Classification of Diseases (ICD) mortality coding.

Additionally, in our empirical analysis, we have presented parsimonious evidence, that higher detection rates are associated with saving lives. Our conditional analysis shows, for example, that if the US would have had the same detection rate trajectory as South Korea, about two-thirds of the reported deaths could have been avoided (about 85,000 lives).

We find that detection rates at the very early stages of the pandemic seem to explain the great divergence in terms of deaths between countries. Moreover, we showed evidence that moving from relatively low to high cumulative detection rates (and thus saving lives) is unlikely and difficult. This is probably due to the high level of efforts needed to expand testing relative to the exponentially growing infections at the early and middle stages of the pandemic. Thus, from the public health point of view, it is better to deploy testing efforts at the first stages of the pandemic process. To do this would be much more advantageous, in terms of saved lives, but also it would be technically, and economically feasible.

Already, many developed countries with well-developed health sectors were not able to avoid unnecessary deaths by their inaction in terms of promoting mass testing to counter the pandemic outbreak at early stages.

To achieve the goal of implementing mass testing from the very beginning of the pandemic outbreak, governments need to understand the consequences of not doing that. Thus, the evidence presented in this paper offers a rigorous macro-level linkage between detection rates and the cumulative number of deaths which may be useful in future pandemics. This evidence also supports the implementation of mass testing in the likely coming secondary pandemic outbreak (so-called second waves).

Further research should be devoted to understanding why the detection capacity in many advanced countries was too weak, late, and also so weakly correlated (if correlated) with the income levels. In this paper, we claim that governments have incentives against test because the public opinion tends to primarily react to the report of the cumulative and the marginal numbers of detected (reported) cases. The contradiction is that something good, such as the increase in the testing efforts by governments, can be perceived by the general public as something negative (due to the increase in detections). In consequence, are low detection rates in developed countries simply a management failure, or are there long-run incentives that promoted this behavior among many rich countries? It is clear that during the ongoing pandemic, improving detection rates is a race against time, but are there institutional and/or technological constraints that hamper detection improvements that can save lives? All these questions are relevant for this and future pandemics. This study claims that all countries in the world should be able to respond to a pandemic outbreak with massive testing in the very short run. This would be an efficient approach since it is also likely that higher detection rates are also associated with a lesser impact of the pandemic on the economy.

## Data Availability Statement

The raw data supporting the conclusions of this article will be made available by the authors, without undue reservation.

## Author Contributions

CV conceived this research, performed the background work, collected the data, performed all statistical analyses, and wrote the paper.

## Conflict of Interest

The author declares that the research was conducted in the absence of any commercial or financial relationships that could be construed as a potential conflict of interest.

## Footnotes

^1^Some evidence has been found claiming that elderly and male individuals are in higher relative risk since the consequences of COVID-19 are more severe amongst them (16–20).

^2^Death-related statistics are nor free of problems. It is recognized that not all deaths due to COVID-19 in all countries are reported following WHO international norms and standards for medical certificates of COVID-19 cause of death and International Classification of Diseases (ICD) mortality coding (29). Moreover, in many countries, there is controversy over whether the COVID-19 death figures are reliable or not (for instance in Spain, Chile, and the UK), especially when these figures are compared against those from the number of excess deaths during the pandemic. More generally, it is a matter of concern that the official accumulated death figures show significant breaks responding probably to counting issues rather than to real deaths' dynamics. In our data, these breaks can be found in Spain, Chile, China, Ecuador, the Philippines, and the United Kingdom.

^3^Available online at: https://ourworldindata.org/coronavirus-source-data

^4^United Nations, Department of Economic and Social Affairs, Population Division. *World Population Prospects 2019*, Online Edition. Rev. 1. United Nations (2020). Available online at: https://population.un.org/wpp/Download/Standard/Population/ (accessed April 19, 2020).

^5^Following Verity et al. (26), the estimated IFRs correct for many types of bias. The infection fatality ratios were obtained after combining adjusted case fatality ratios with data on infection prevalence amongst individuals returning home from Wuhan in repatriation flights.

^6^https://data.worldbank.org/indicator/ (accessed April 24, 2020).

^7^https://apps.who.int/gho/data/node.main.A830?lang=en (accessed April 24, 2020).

^8^Differently to Bommer and Vollmer (32), we include the incubation period while avoiding the subtraction of the number of days between the onset of symptoms and detection to the relevant lag period. These differences explain the discrepancies between both set of estimates. Moreover, by combining the cumulative distribution function of the SARS-CoV-2 incubation period as reported in Lauer et al. (31) and an approximation of the Gamma distribution with correction for epidemic growth of the days between the onset of symptoms to death as reported in Verity et al. (26), one can calculate a vector of probabilities to weight the cumulative number of deaths required in equation 1. The weighting vector goes from *t*_−2_ (representing the proportion of deaths of those who experienced 1 day between infection and the onset of symptoms, plus one day from the onset of symptoms to death) to *t*_−72_ (representing the proportion of deaths of those who experienced 12 days between infection and the onset of symptoms and 60 days between the onset of symptoms to death). The smoothed approach produces almost an identical estimation of the cumulative number of infected individuals. Given that and for the sake of simplicity, we prefer to use the non-smoothing approach.

^9^For instance, the reported IFRs for a group of 9,496 Danish blood donors with no comorbidity aged 17–69 reached 0.082% (35).

^10^In the context of the pandemic, a synchronic estimation refers to the use of information of countries in the same pandemic phase, that is, after the same number of days since the pandemic outbreak. On the contrary, a non-synchronic estimation neglects the pandemic phases but considers as reference period the calendar day.

^11^Output tables without the square of the detection rates are available in the Supplementary Material.

^12^In constant 2017 international dollars with the same purchase power.

^13^https://apps.who.int/gho/data/node.main.A830?lang=en (accessed April 24, 2020).

## Supplementary Material

**Supplementary Set of Figures**. Estimated number of SARS-CoV-2 infections by country.

Supplementary Table 1 Synchronic multiple linear regression of the natural logarithm of the cumulative number of deaths on the estimated detections rates (linear specification).

Supplementary Table 2 Synchronic multiple linear regression of the natural logarithm of the cumulative number of deaths on the estimated number of SARS-CoV-2 infections.

Supplementary Table 3 Synchronic robust multiple linear regression of the natural logarithm of the cumulative number of deaths on the estimated detections rates (linear specification).

Supplementary Table 4 Synchronic robust multiple linear regression of the natural logarithm of the cumulative number of deaths on the estimated detections rates (non-linear specification).

Supplementary Table 5 Synchronic multiple linear regression of the natural logarithm of the cumulative number of deaths on the estimated number of SARS-CoV-2 infections.
