# Supplementary material for: SARS-CoV-2 Infections in the World: An Estimation of the Infected Population and a Measure of How Higher Detection Rates Save Lives
Source: Front Public Health. 2020 Sep 25;8:489. doi: 10.3389/fpubh.2020.00489 (PMC7545403; doi:10.3389/fpubh.2020.00489)
Supplement: Supplementary Table 1 — Synchronic multiple linear regression of the natural logarithm of the cumulative number of deaths on the estimated detections rates (linear specification). [file Data_Sheet_2.docx]

**SUPPLEMENTARY TABLE 1 | Synchronic multiple linear regression of the natural logarithm of the cumulative number of deaths on the estimated detections rates (linear specification).**

| Dependent Variable: Ln(deaths) /  Explanatory Variables | Days since the first 100 cases were confirmed | | | | | | | |
| --- | --- | --- | --- | --- | --- | --- | --- | --- |
|  | 15 | | 60 | | | 105 | | |
|  | Model (1) | Model (2) | Model (3) | Model (4) | Model (5) | Model (6) | Model (7) | Model (8) |
| Estimated detection rate | -0.0637** | -0.0646 | -0.0500*** | -0.0489*** |  | -0.0483*** | -0.0466*** |  |
|  | (0.0276) | (0.0391) | (0.00883) | (0.00874) |  | (0.00613) | (0.00632) |  |
| Estimated detection rate 15 days after PO |  |  |  |  | -6.414** |  |  | -4.897** |
|  |  |  |  |  | (2.842) |  |  | (2.207) |
| Infection fatality rate | 0.568 | 0.497 | 1.212*** | 1.150*** | 0.991** | 1.287*** | 1.274*** | 1.176*** |
|  | (0.375) | (0.383) | (0.361) | (0.358) | (0.436) | (0.241) | (0.246) | (0.373) |
| Population size (Ln) | -0.0634 | -0.0546 | 0.0448 | 0.0553 | 0.0868 | 0.114*** | 0.106** | 0.0914 |
|  | (0.0697) | (0.0736) | (0.0926) | (0.0855) | (0.0966) | (0.0431) | (0.0440) | (0.0615) |
| Confirmed cases (Ln) | 0.920*** | 0.855*** | 0.905*** | 0.866*** | 0.764*** | 0.910*** | 0.909*** | 0.883*** |
|  | (0.100) | (0.145) | (0.0817) | (0.0761) | (0.103) | (0.0389) | (0.0397) | (0.0716) |
| GDP per capita (Ln) | -0.454*** | -0.420*** | 0.0273 | 0.0533 | 0.0609 | 0.156* | 0.142 | -0.00509 |
|  | (0.111) | (0.117) | (0.105) | (0.115) | (0.158) | (0.0792) | (0.0891) | (0.172) |
| Health spending as % of GDP | -0.0383 | -0.0433 | 0.0141 | -0.00102 | 0.0110 | 0.0223 | 0.00582 | 0.00460 |
|  | (0.0351) | (0.0391) | (0.0257) | (0.0292) | (0.0331) | (0.0208) | (0.0232) | (0.0368) |
| BCG group 2 |  | -0.121 |  | 0.256 | 0.417 |  | 0.177 | 0.425 |
|  |  | (0.392) |  | (0.173) | (0.276) |  | (0.141) | (0.280) |
| BCG group 3 |  | -0.0294 |  | 0.130 | 0.309 |  | 0.210 | 0.477 |
|  |  | (0.420) |  | (0.291) | (0.387) |  | (0.218) | (0.414) |
| BCG group 4 |  | -0.378 |  | -0.123 | 0.0931 |  | -0.0725 | 0.0842 |
|  |  | (0.426) |  | (0.165) | (0.273) |  | (0.120) | (0.241) |
| BCG group 5 |  | -0.330 |  | -0.176 | -0.126 |  | -0.0299 | 0.0791 |
|  |  | (0.283) |  | (0.148) | (0.195) |  | (0.110) | (0.186) |
| Constant | 2.445 | 2.745 | -3.591** | -3.476** | -3.524* | -6.159*** | -5.787*** | -4.360** |
|  | (1.552) | (1.947) | (1.569) | (1.649) | (2.010) | (0.871) | (1.035) | (1.696) |
| Observations | 87 | 87 | 84 | 84 | 84 | 74 | 74 | 74 |
| R-squared | 0.598 | 0.616 | 0.928 | 0.934 | 0.902 | 0.974 | 0.976 | 0.937 |
| R-squared adjusted | 0.568 | 0.565 | 0.923 | 0.925 | 0.889 | 0.972 | 0.972 | 0.927 |
| F-test | 28.57 | 17.79 | 207.8 | 200.1 | 76.28 | 367.6 | 248.6 | 99.38 |

*Note: Standard errors in parentheses. Significance levels: *** p<0.01, ** p<0.05, * p<0.1. Source: Own elaboration.*

**SUPPLEMENTARY TABLE 2 | Synchronic multiple linear regression of the natural logarithm of the cumulative number of deaths on the estimated number of SARS-CoV-2 infections.**

| Dependent Variable: Ln(deaths) /  Explanatory Variables | Days since the first 100 cases were confirmed | | | | | | |
| --- | --- | --- | --- | --- | --- | --- | --- |
|  | 15 | | 60 | | 105 | |  |
|  | Model (1) | Model (2) | Model (3) | Model (4) | Model (5) | Model (6) |  |
| Estimated Infections (in Ln) | 0.696*** | 0.710*** | 0.950*** | 0.941*** | 0.950*** | 0.943*** |  |
|  | (0.0606) | (0.0700) | (0.0489) | (0.0453) | (0.0250) | (0.0256) |  |
| Infection fatality rate | 0.960*** | 0.826*** | 1.629*** | 1.540*** | 1.632*** | 1.611*** |  |
|  | (0.302) | (0.280) | (0.244) | (0.241) | (0.147) | (0.150) |  |
| Population size (Ln) | -0.148** | -0.150** | -0.0297 | -0.0340 | 0.0595* | 0.0572 |  |
|  | (0.0722) | (0.0751) | (0.0757) | (0.0681) | (0.0351) | (0.0352) |  |
| GDP per capita (Ln) | -0.336*** | -0.368*** | 0.115* | 0.106* | 0.175*** | 0.171*** |  |
|  | (0.0925) | (0.0880) | (0.0602) | (0.0602) | (0.0497) | (0.0512) |  |
| Health spending as % of GDP | -0.0676** | -0.0594* | -0.00523 | -0.0186 | 0.00274 | -0.0105 |  |
|  | (0.0291) | (0.0309) | (0.0192) | (0.0231) | (0.0126) | (0.0143) |  |
| BCG group 2 |  | -0.470 |  | 0.0549 |  | 0.131 |  |
|  |  | (0.341) |  | (0.110) |  | (0.0866) |  |
| BCG group 3 |  | -0.565** |  | -0.0550 |  | 0.143 |  |
|  |  | (0.250) |  | (0.181) |  | (0.114) |  |
| BCG group 4 |  | -0.685*** |  | -0.362*** |  | -0.0958 |  |
|  |  | (0.255) |  | (0.136) |  | (0.105) |  |
| BCG group 5 |  | -0.432** |  | -0.248** |  | -0.0392 |  |
|  |  | (0.208) |  | (0.121) |  | (0.0760) |  |
| Constant | 1.499 | 2.164 | -6.693*** | -6.096*** | -8.711*** | -8.440*** |  |
|  | (1.286) | (1.343) | (0.974) | (1.016) | (0.602) | (0.663) |  |
| Observations | 87 | 87 | 84 | 84 | 74 | 74 |  |
| R-squared | 0.646 | 0.687 | 0.962 | 0.969 | 0.985 | 0.986 |  |
| R-squared adjusted | 0.624 | 0.651 | 0.960 | 0.965 | 0.984 | 0.985 |  |
| F-test | 31.86 | 20.74 | 588.4 | 409.1 | 1247 | 648.4 |  |

*Note: Standard errors in parentheses. Significance levels: *** p<0.01, ** p<0.05, * p<0.1. Source: Own elaboration.*

**SUPPLEMENTARY TABLE 3 | Synchronic robust multiple linear regression of the natural logarithm of the cumulative number of deaths on the estimated detections rates (linear specification).**

| Dependent Variable: Ln(deaths) /  Explanatory Variables | Days since the first 100 cases were confirmed | | | | | | | |
| --- | --- | --- | --- | --- | --- | --- | --- | --- |
|  | 15 | | 60 | | | 105 | | |
|  | Model (1) | Model (2) | Model (3) | Model (4) | Model (5) | Model (6) | Model (7) | Model (8) |
| Estimated detection rate | -0.0730*** | -0.104*** | -0.0498*** | -0.0485*** |  | -0.0510*** | -0.0478*** |  |
|  | (0.0133) | (0.0132) | (0.00458) | (0.00462) |  | (0.00310) | (0.00323) |  |
| Estimated detection rate 15 days after PO |  |  |  |  | -8.385*** |  |  | -5.142*** |
|  |  |  |  |  | (1.144) |  |  | (1.186) |
| Infection fatality rate | 0.669* | 0.742** | 1.240*** | 1.105*** | 1.082*** | 1.070*** | 1.101*** | 1.169*** |
|  | (0.353) | (0.337) | (0.203) | (0.198) | (0.271) | (0.143) | (0.143) | (0.285) |
| Population size (Ln) | -0.0557 | -0.0656 | 0.0954* | 0.0907* | 0.0905 | 0.0812** | 0.0614* | 0.0598 |
|  | (0.0693) | (0.0656) | (0.0500) | (0.0486) | (0.0671) | (0.0328) | (0.0328) | (0.0659) |
| Confirmed cases (Ln) | 0.920*** | 0.743*** | 0.880*** | 0.870*** | 0.739*** | 0.937*** | 0.945*** | 0.888*** |
|  | (0.119) | (0.119) | (0.0515) | (0.0515) | (0.0769) | (0.0315) | (0.0315) | (0.0696) |
| GDP per capita (Ln) | -0.455*** | -0.386*** | 0.0961 | 0.114 | 0.132 | 0.266*** | 0.254*** | 0.00917 |
|  | (0.126) | (0.121) | (0.0792) | (0.0790) | (0.112) | (0.0580) | (0.0587) | (0.116) |
| Health spending as % of GDP | -0.0338 | -0.0405 | 0.0310 | 0.0222 | -0.000157 | 0.00798 | -0.0126 | -0.00851 |
|  | (0.0357) | (0.0356) | (0.0207) | (0.0212) | (0.0289) | (0.0156) | (0.0170) | (0.0339) |
| BCG group 2 |  | -0.268 |  | 0.159 | 0.364 |  | 0.185 | 0.477** |
|  |  | (0.293) |  | (0.170) | (0.232) |  | (0.118) | (0.233) |
| BCG group 3 |  | -0.311 |  | 0.339 | 0.366 |  | 0.341* | 0.494 |
|  |  | (0.355) |  | (0.207) | (0.282) |  | (0.182) | (0.359) |
| BCG group 4 |  | -0.794*** |  | -0.135 | -0.0841 |  | 0.0493 | 0.0956 |
|  |  | (0.248) |  | (0.143) | (0.192) |  | (0.105) | (0.207) |
| BCG group 5 |  | -0.504** |  | -0.0135 | -0.0671 |  | 0.00174 | 0.0304 |
|  |  | (0.217) |  | (0.124) | (0.170) |  | (0.0867) | (0.172) |
| Constant | 2.287 | 3.630** | -5.017*** | -4.873*** | -3.893*** | -6.631*** | -6.263*** | -3.883*** |
|  | (1.450) | (1.407) | (1.008) | (1.005) | (1.382) | (0.708) | (0.731) | (1.437) |
| Observations | 87 | 87 | 84 | 84 | 84 | 74 | 74 | 74 |
| R-squared | 0.606 | 0.695 | 0.952 | 0.957 | 0.922 | 0.982 | 0.983 | 0.931 |
| R-squared adjusted | 0.577 | 0.655 | 0.948 | 0.952 | 0.911 | 0.980 | 0.980 | 0.920 |
| F-test | 20.54 | 17.36 | 254.9 | 164.1 | 86.05 | 603.1 | 364.6 | 85.06 |

*Note: Standard errors in parentheses. Significance levels: *** p<0.01, ** p<0.05, * p<0.1. Source: Own elaboration.*

**SUPPLEMENTARY TABLE 4 | Synchronic robust multiple linear regression of the natural logarithm of the cumulative number of deaths on the estimated detections rates (non-linear specification).**

| Dependent Variable: Ln(deaths) /  Explanatory Variables | Days since the first 100 cases were confirmed | | | | | | | |
| --- | --- | --- | --- | --- | --- | --- | --- | --- |
|  | 15 | | 60 | | | 105 | | |
|  | Model (1) | Model (2) | Model (3) | Model (4) | Model (5) | Model (6) | Model (7) | Model (8) |
| Estimated detection rate | -0.196*** | -0.224*** | -0.133*** | -0.139*** |  | -0.111*** | -0.111*** |  |
|  | (0.0338) | (0.0365) | (0.0145) | (0.0141) |  | (0.0100) | (0.0116) |  |
| Estimated detection rate (Squared) | 0.00410*** | 0.00488*** | 0.00182*** | 0.00194*** |  | 0.00127*** | 0.00128*** |  |
|  | (0.00100) | (0.00106) | (0.000322) | (0.000310) |  | (0.000206) | (0.000237) |  |
| Estimated detection rate 15 days after PO |  |  |  |  | -24.68*** |  |  | -21.28*** |
|  |  |  |  |  | (2.969) |  |  | (3.299) |
| Estimated detection rate 15 days after PO (squared) |  |  |  |  | 64.62*** |  |  | 56.79*** |
|  |  |  |  |  | (11.02) |  |  | (12.22) |
| Infection fatality rate | 1.063*** | 1.028*** | 1.590*** | 1.505*** | 1.243*** | 1.291*** | 1.372*** | 1.321*** |
|  | (0.334) | (0.333) | (0.171) | (0.162) | (0.209) | (0.119) | (0.137) | (0.232) |
| Population size (Ln) | -0.124* | -0.122* | 0.0342 | 0.0388 | 0.0348 | 0.0792*** | 0.0760** | 0.0141 |
|  | (0.0663) | (0.0654) | (0.0415) | (0.0390) | (0.0505) | (0.0269) | (0.0309) | (0.0534) |
| Confirmed cases (Ln) | 0.873*** | 0.769*** | 0.896*** | 0.879*** | 0.732*** | 0.918*** | 0.916*** | 0.833*** |
|  | (0.109) | (0.114) | (0.0418) | (0.0404) | (0.0571) | (0.0254) | (0.0293) | (0.0551) |
| GDP per capita (Ln) | -0.459*** | -0.421*** | 0.106 | 0.124** | 0.204** | 0.250*** | 0.219*** | 0.0859 |
|  | (0.114) | (0.115) | (0.0644) | (0.0619) | (0.0823) | (0.0470) | (0.0547) | (0.0912) |
| Health spending as % of GDP | -0.0529 | -0.0483 | 0.0145 | 0.00966 | -0.00182 | 0.0122 | 0.00277 | 0.00960 |
|  | (0.0326) | (0.0340) | (0.0171) | (0.0168) | (0.0214) | (0.0128) | (0.0159) | (0.0268) |
| BCG group 2 |  | -0.306 |  | 0.0181 | 0.116 |  | 0.116 | 0.123 |
|  |  | (0.287) |  | (0.135) | (0.175) |  | (0.109) | (0.189) |
| BCG group 3 |  | -0.370 |  | 0.125 | 0.228 |  | 0.132 | 0.186 |
|  |  | (0.347) |  | (0.163) | (0.212) |  | (0.168) | (0.288) |
| BCG group 4 |  | -0.712*** |  | -0.278** | -0.0546 |  | -0.0172 | 0.0390 |
|  |  | (0.246) |  | (0.114) | (0.150) |  | (0.0966) | (0.172) |
| BCG group 5 |  | -0.400* |  | -0.139 | 0.0472 |  | 0.0222 | 0.0943 |
|  |  | (0.207) |  | (0.0985) | (0.128) |  | (0.0804) | (0.139) |
| Constant | 4.064*** | 4.874*** | -3.780*** | -3.633*** | -3.192*** | -5.947*** | -5.619*** | -2.937** |
|  | (1.409) | (1.450) | (0.828) | (0.799) | (1.063) | (0.574) | (0.680) | (1.196) |
| Observations | 87 | 87 | 83 | 83 | 83 | 73 | 73 | 73 |
| R-squared | 0.677 | 0.705 | 0.969 | 0.974 | 0.958 | 0.988 | 0.985 | 0.958 |
| R-squared adjusted | 0.649 | 0.662 | 0.966 | 0.970 | 0.952 | 0.987 | 0.983 | 0.950 |
| F-test | 23.67 | 16.31 | 332.5 | 244.9 | 147.3 | 770.1 | 373.1 | 126.1 |

*Note: Standard errors in parentheses. Significance levels: *** p<0.01, ** p<0.05, * p<0.1. Source: Own elaboration.*

**SUPPLEMENTA**

**RY TABLE 5 | Synchronic robust multiple linear regression of the natural logarithm of the cumulative number of deaths on the estimated number of SARS-CoV-2 infections.**

| Dependent Variable: Ln(deaths) /  Explanatory Variables | Days since the first 100 cases were confirmed | | | | | |
| --- | --- | --- | --- | --- | --- | --- |
|  | 15 | | 60 | | 105 | |
|  | Model (1) | Model (2) | Model (1) | Model (2) | Model (1) | Model (2) |
| Estimated Infections | 0.703*** | 0.718*** | 0.912*** | 0.911*** | 0.954*** | 0.948*** |
|  | (0.0705) | (0.0751) | (0.0300) | (0.0305) | (0.0172) | (0.0187) |
| Infection fatality rate | 1.028*** | 0.925*** | 1.694*** | 1.589*** | 1.343*** | 1.332*** |
|  | (0.337) | (0.333) | (0.156) | (0.148) | (0.102) | (0.108) |
| Population size (Ln) | -0.154** | -0.162** | 0.0249 | 0.0223 | 0.0574*** | 0.0492** |
|  | (0.0703) | (0.0686) | (0.0353) | (0.0336) | (0.0215) | (0.0226) |
| GDP per capita (Ln) | -0.350*** | -0.409*** | 0.111** | 0.110** | 0.250*** | 0.257*** |
|  | (0.109) | (0.110) | (0.0498) | (0.0488) | (0.0348) | (0.0369) |
| Health spending as % of GDP | -0.0679* | -0.0620* | 0.0124 | 0.00539 | -0.000305 | -0.0117 |
|  | (0.0345) | (0.0353) | (0.0158) | (0.0156) | (0.0111) | (0.0124) |
| BCG group 2 |  | -0.417 |  | 0.0174 |  | 0.138 |
|  |  | (0.290) |  | (0.125) |  | (0.0850) |
| BCG group 3 |  | -0.628* |  | 0.0533 |  | 0.144 |
|  |  | (0.352) |  | (0.153) |  | (0.133) |
| BCG group 4 |  | -0.797*** |  | -0.292*** |  | 0.0563 |
|  |  | (0.236) |  | (0.104) |  | (0.0768) |
| BCG group 5 |  | -0.486** |  | -0.128 |  | -0.00109 |
|  |  | (0.207) |  | (0.0898) |  | (0.0633) |
| Constant | 1.600 | 2.664* | -7.272*** | -6.976*** | -9.158*** | -8.974*** |
|  | (1.349) | (1.367) | (0.643) | (0.636) | (0.446) | (0.481) |
| Observations | 87 | 87 | 84 | 84 | 74 | 74 |
| R-squared | 0.624 | 0.674 | 0.971 | 0.976 | 0.991 | 0.991 |
| R-squared adjusted | 0.600 | 0.636 | 0.969 | 0.973 | 0.990 | 0.989 |
| F-test | 26.84 | 17.71 | 521.8 | 334.2 | 1455 | 745.7 |

*Note: Standard errors in parentheses. Significance levels: *** p<0.01, ** p<0.05, * p<0.1. Source: Own elaboration.*
