# Supplementary figures and images for: SARS-CoV-2 Infections in the World: An Estimation of the Infected Population and a Measure of How Higher Detection Rates Save Lives
Source: Front Public Health. 2020 Sep 25;8:489. doi: 10.3389/fpubh.2020.00489 (PMC7545403; doi:10.3389/fpubh.2020.00489)

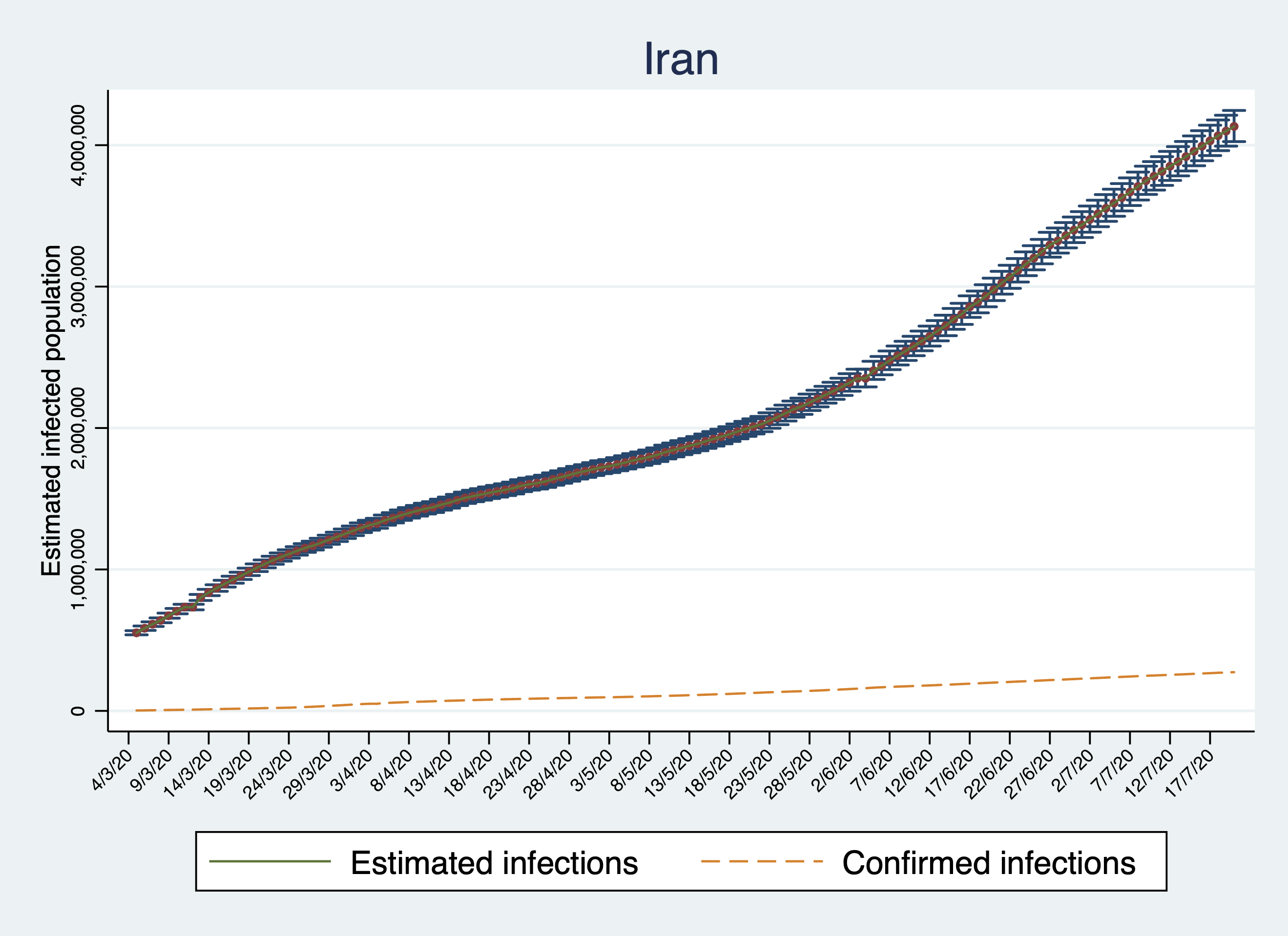

Supplement: Supplementary file 2 [file Data_Sheet_1.ZIP › Country_eni/Iran_20julio.png]

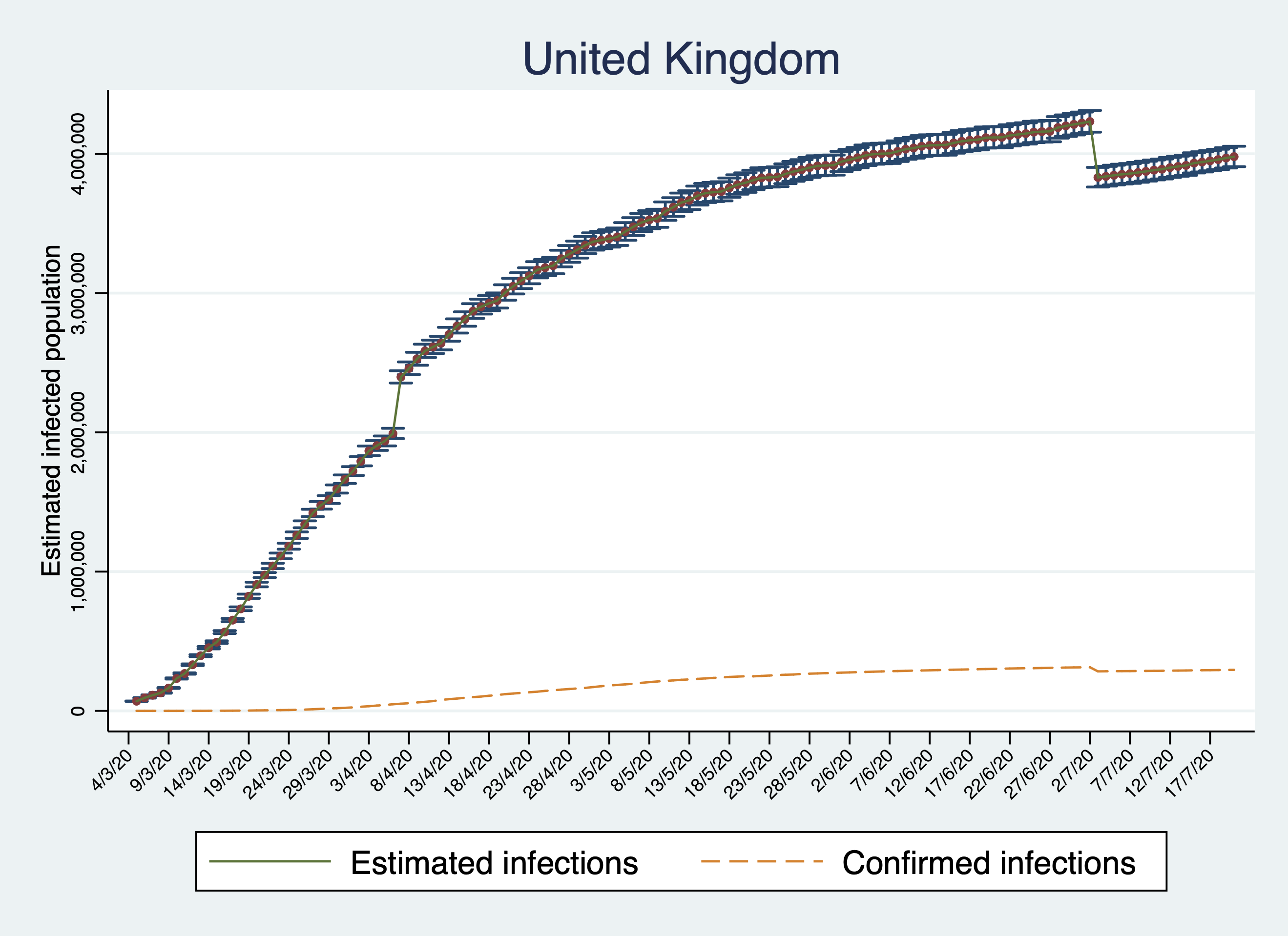

Supplement: Supplementary file 2 [file Data_Sheet_1.ZIP › Country_eni/United Kingdom_20julio.png]

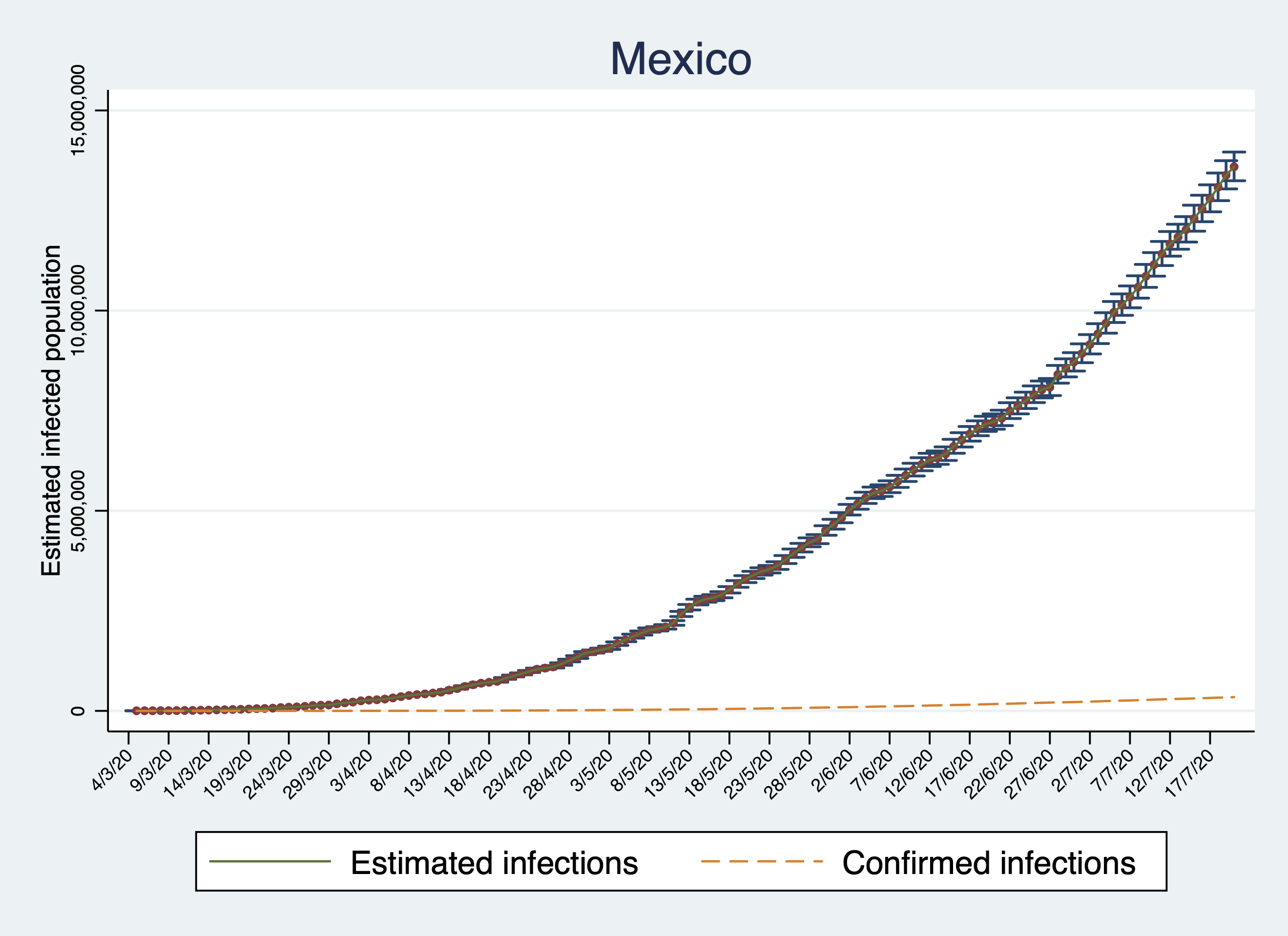

Supplement: Supplementary file 2 [file Data_Sheet_1.ZIP › Country_eni/Mexico_20julio.png]

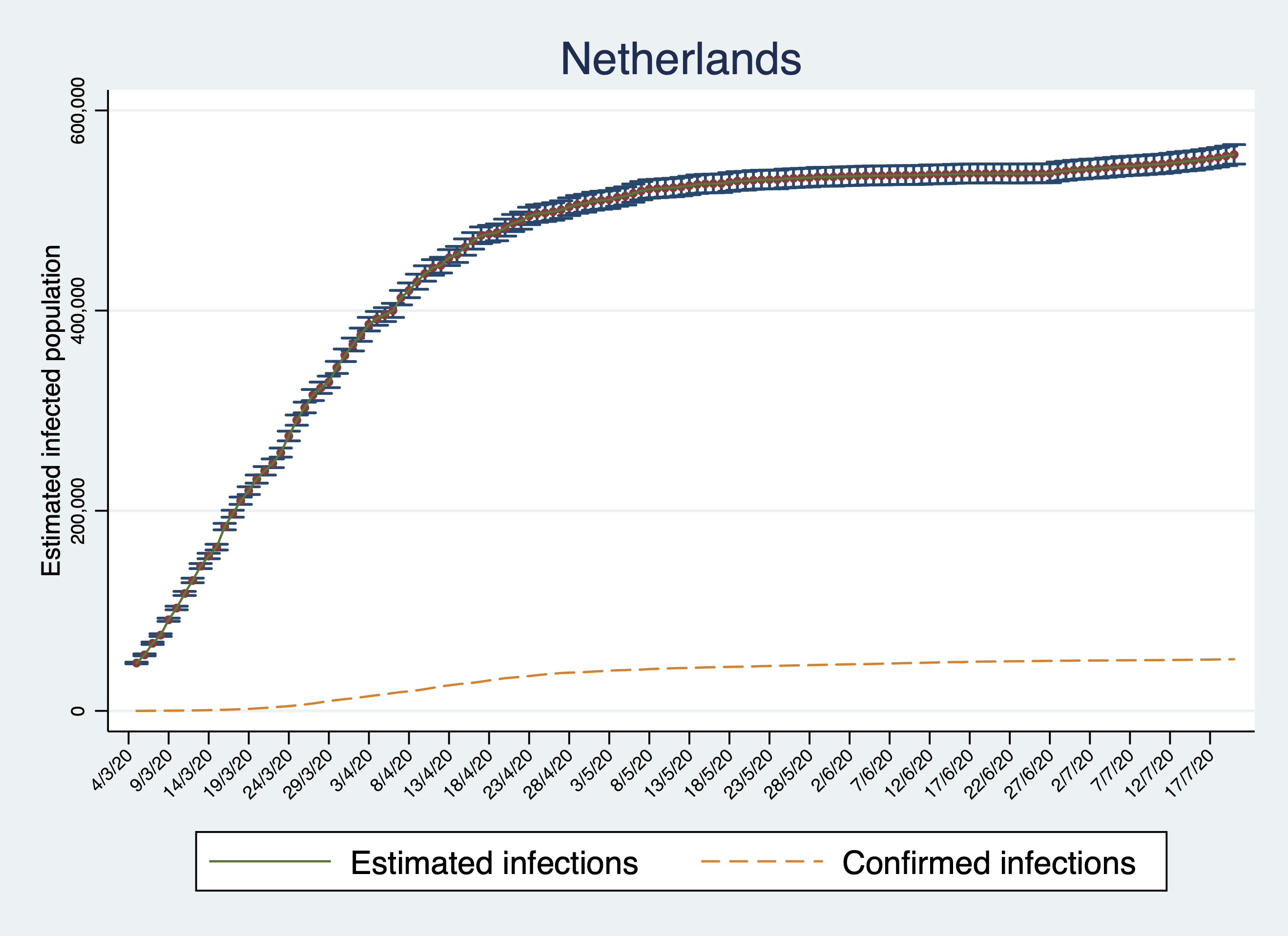

Supplement: Supplementary file 2 [file Data_Sheet_1.ZIP › Country_eni/Netherlands_20julio.png]

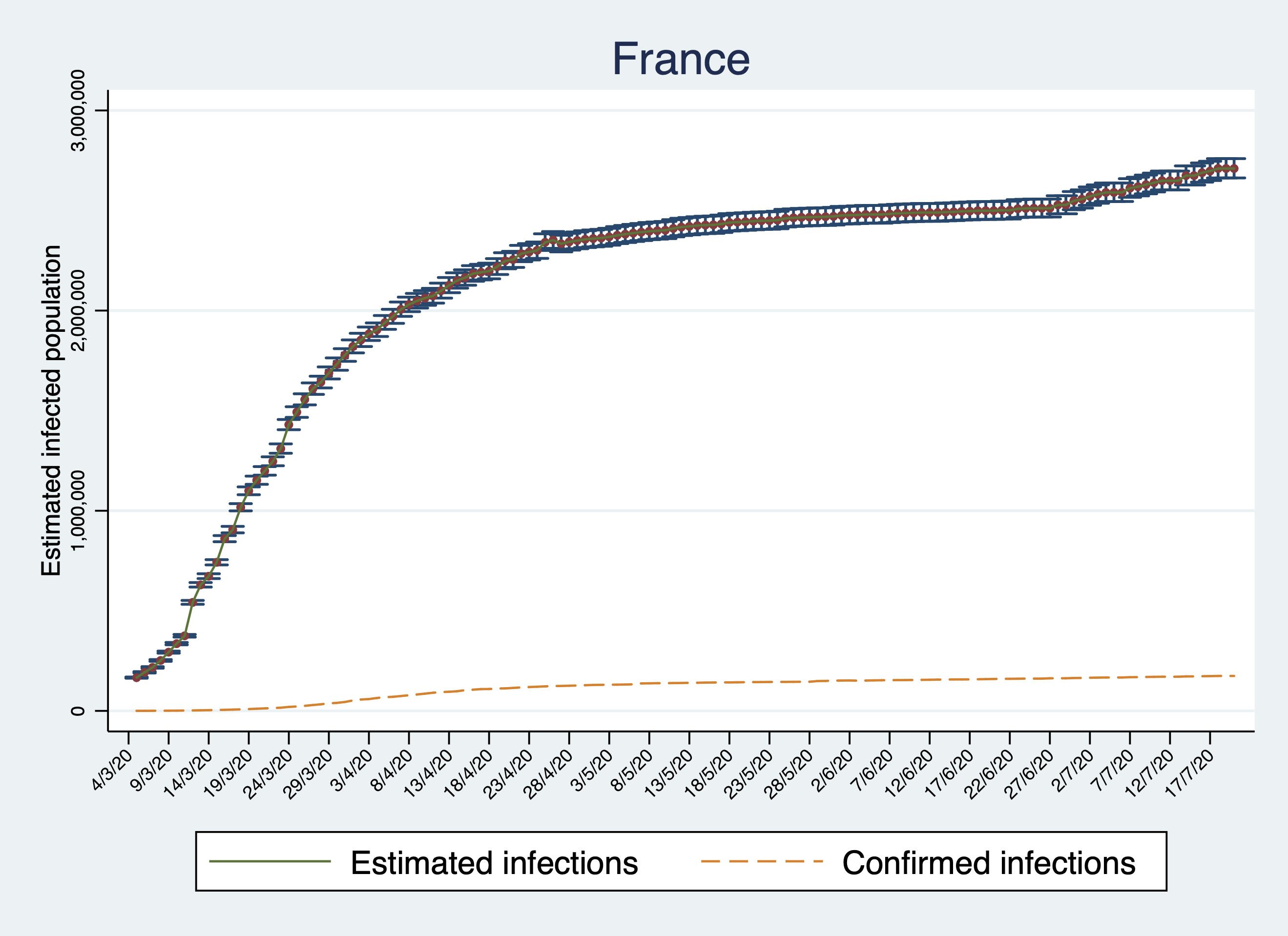

Supplement: Supplementary file 2 [file Data_Sheet_1.ZIP › Country_eni/France_20julio.png]

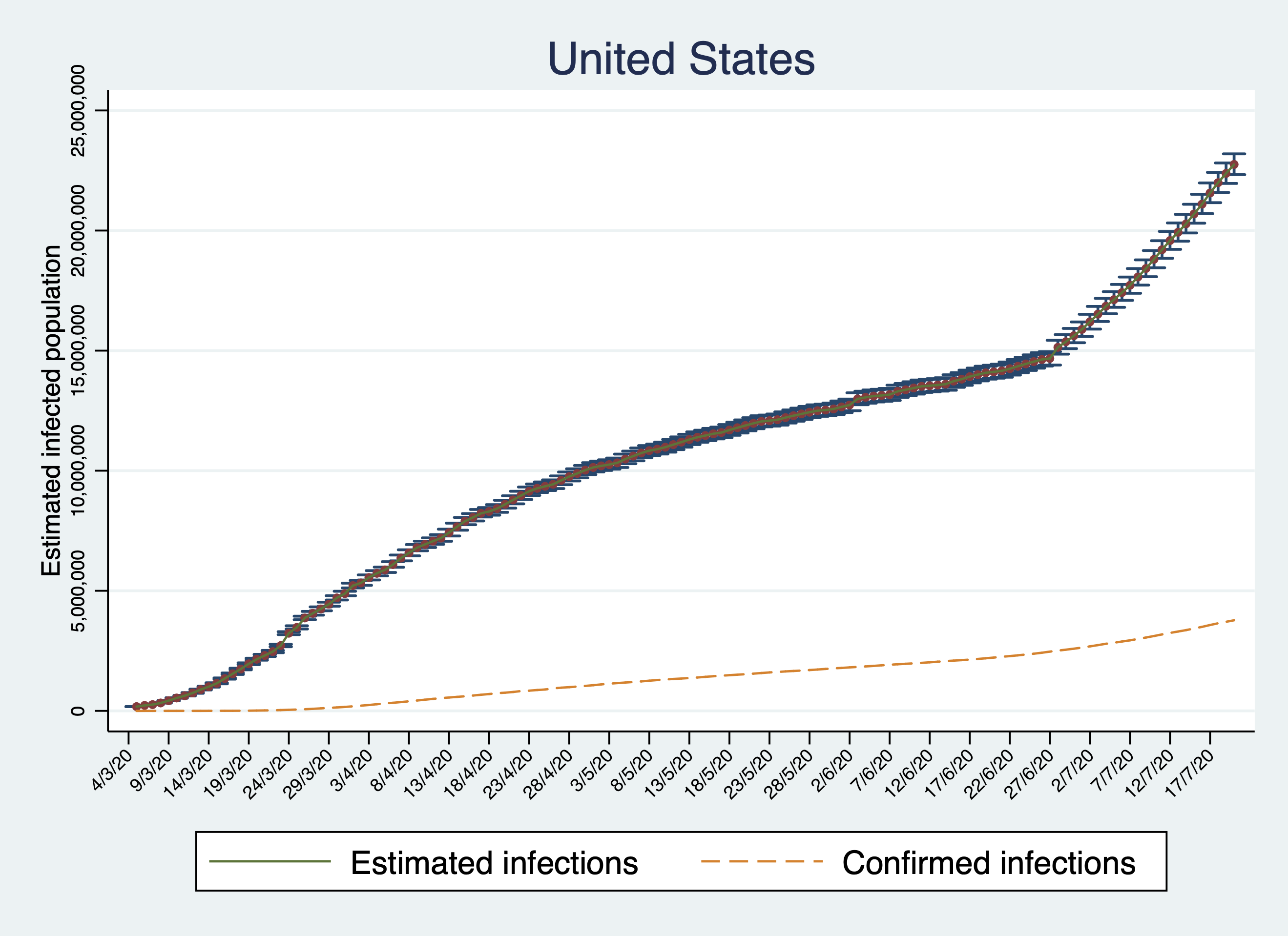

Supplement: Supplementary file 2 [file Data_Sheet_1.ZIP › Country_eni/United States_20julio.png]

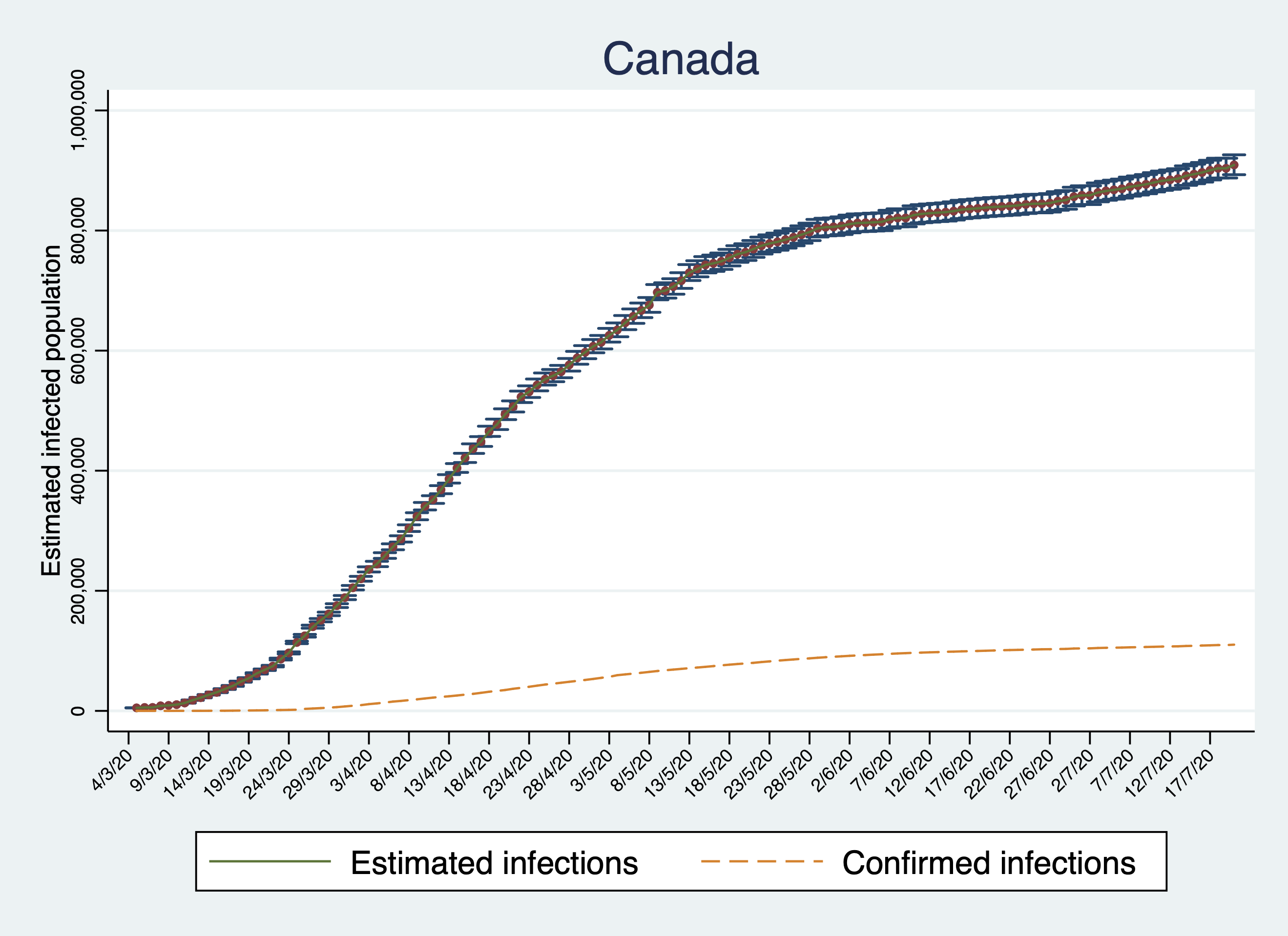

Supplement: Supplementary file 2 [file Data_Sheet_1.ZIP › Country_eni/Canada_20julio.png]

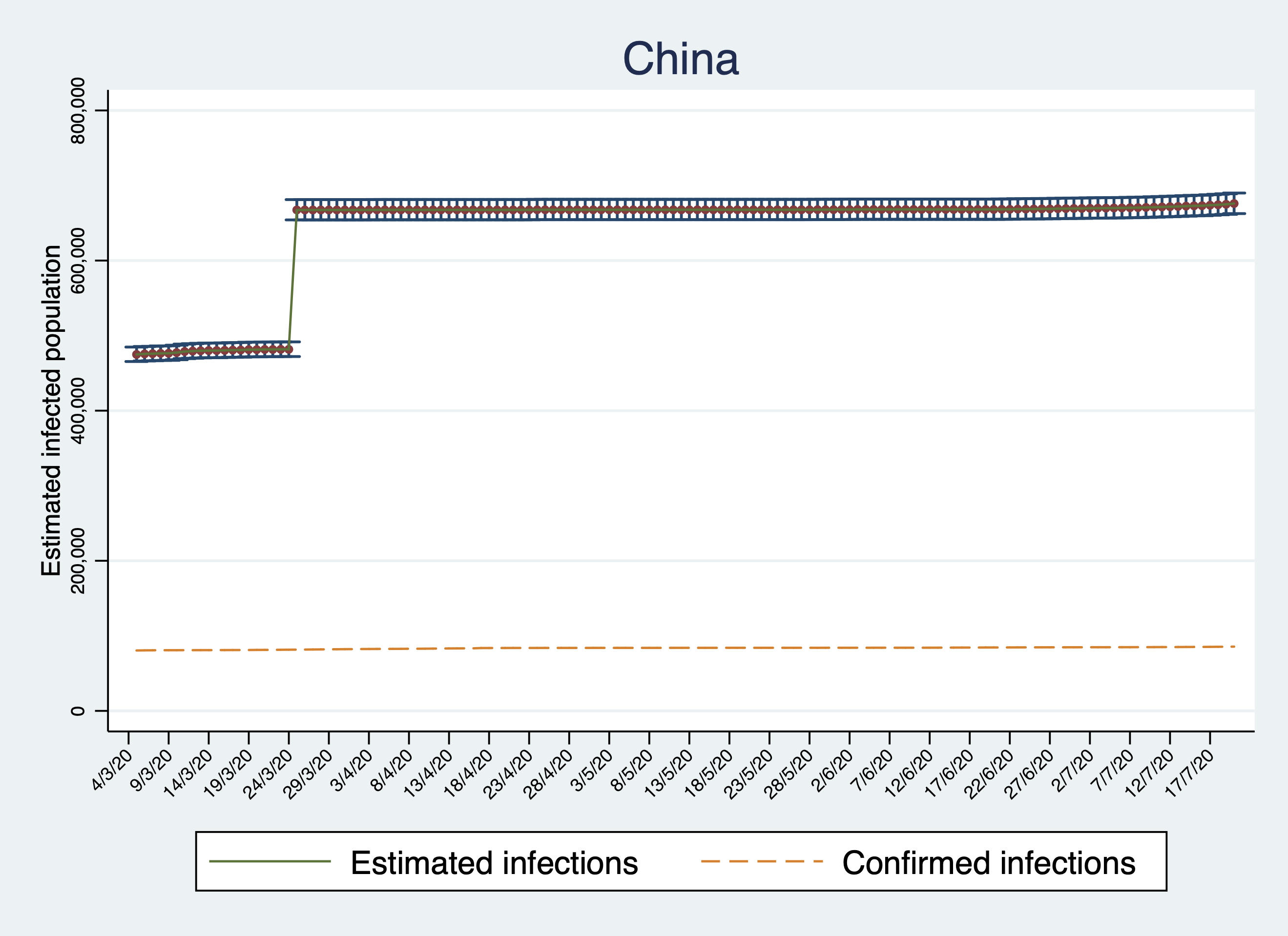

Supplement: Supplementary file 2 [file Data_Sheet_1.ZIP › Country_eni/China_20julio.png]

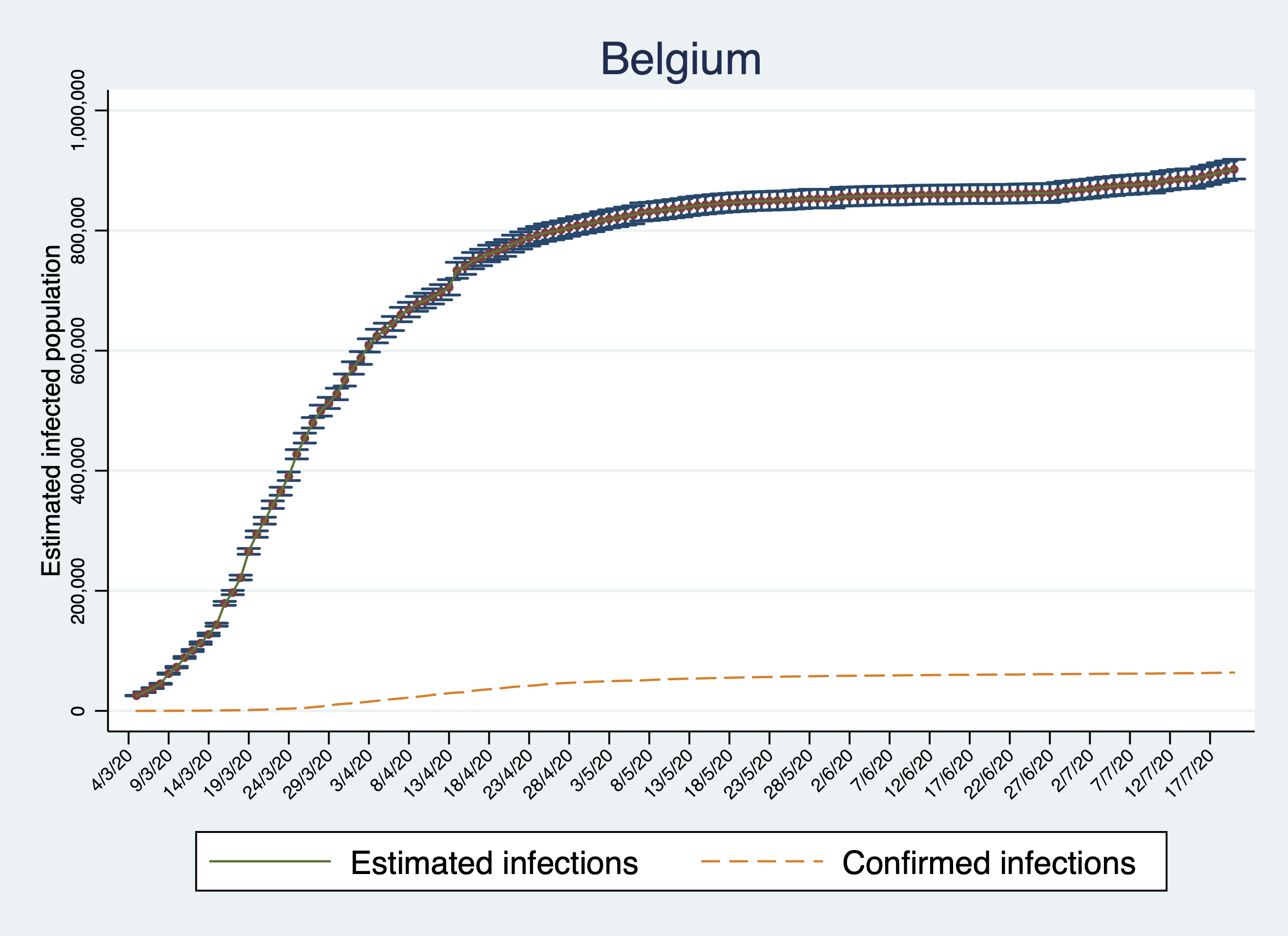

Supplement: Supplementary file 2 [file Data_Sheet_1.ZIP › Country_eni/Belgium_20julio.png]

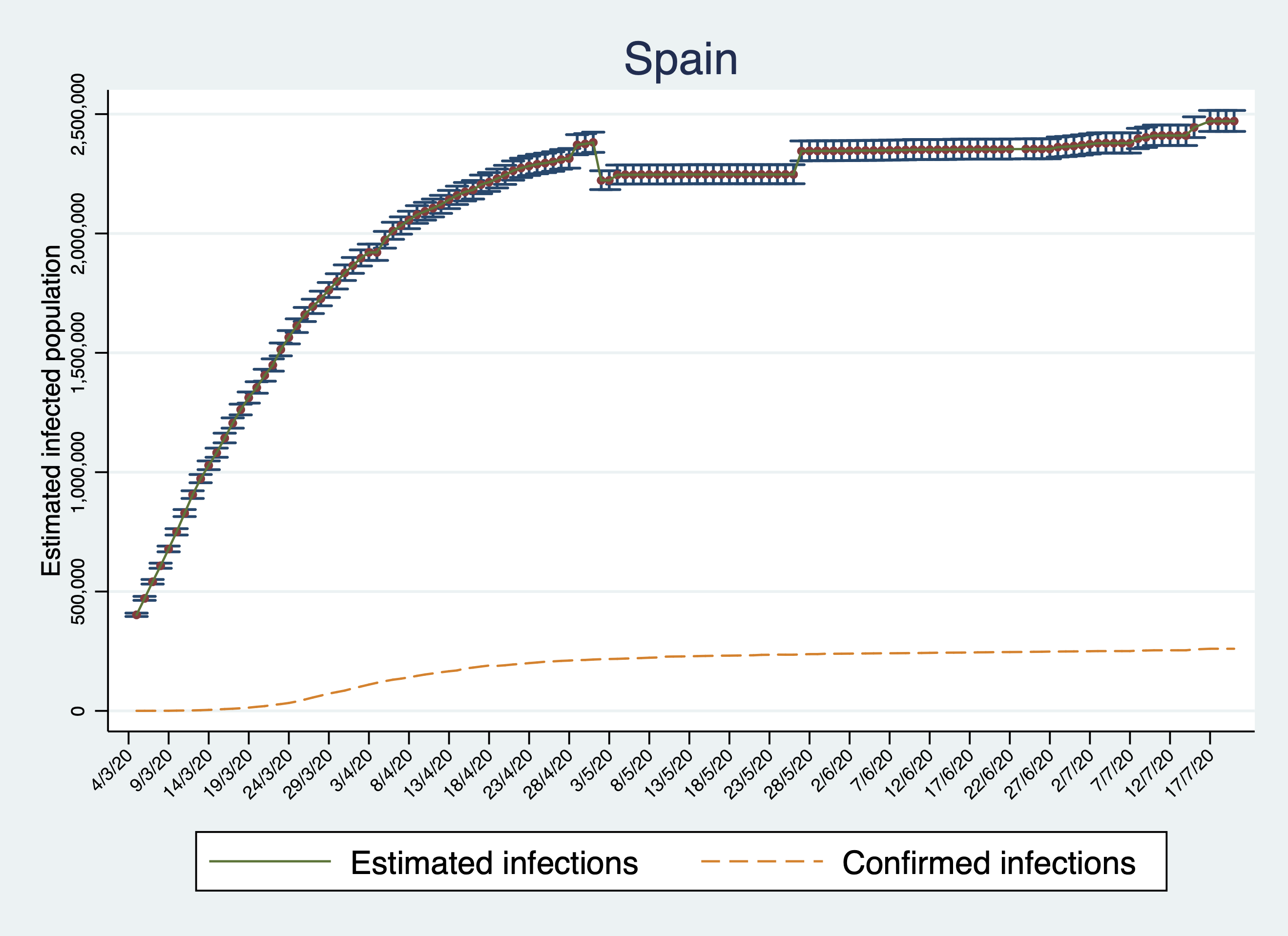

Supplement: Supplementary file 2 [file Data_Sheet_1.ZIP › Country_eni/Spain_20julio.png]

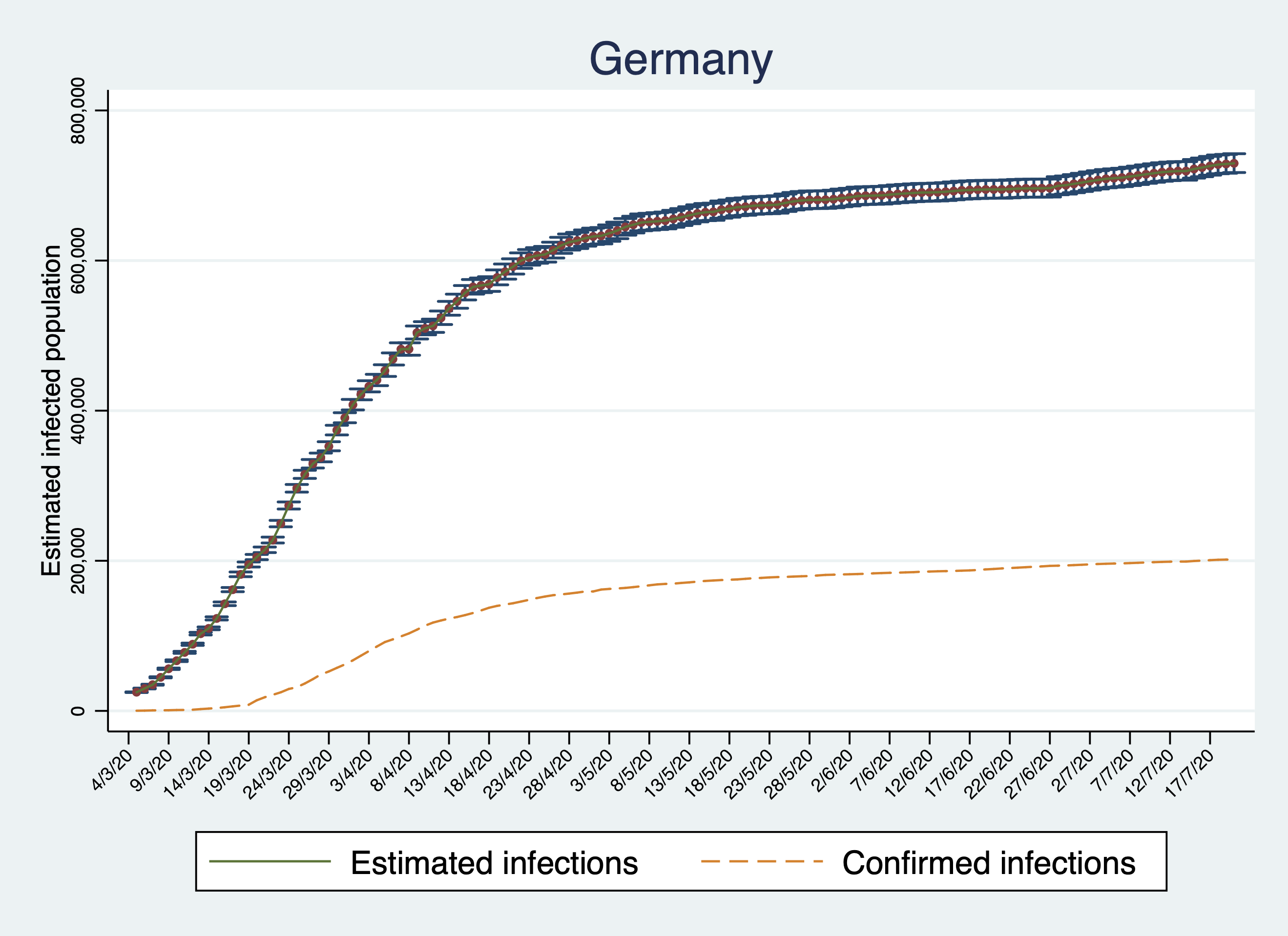

Supplement: Supplementary file 2 [file Data_Sheet_1.ZIP › Country_eni/Germany_20julio.png]

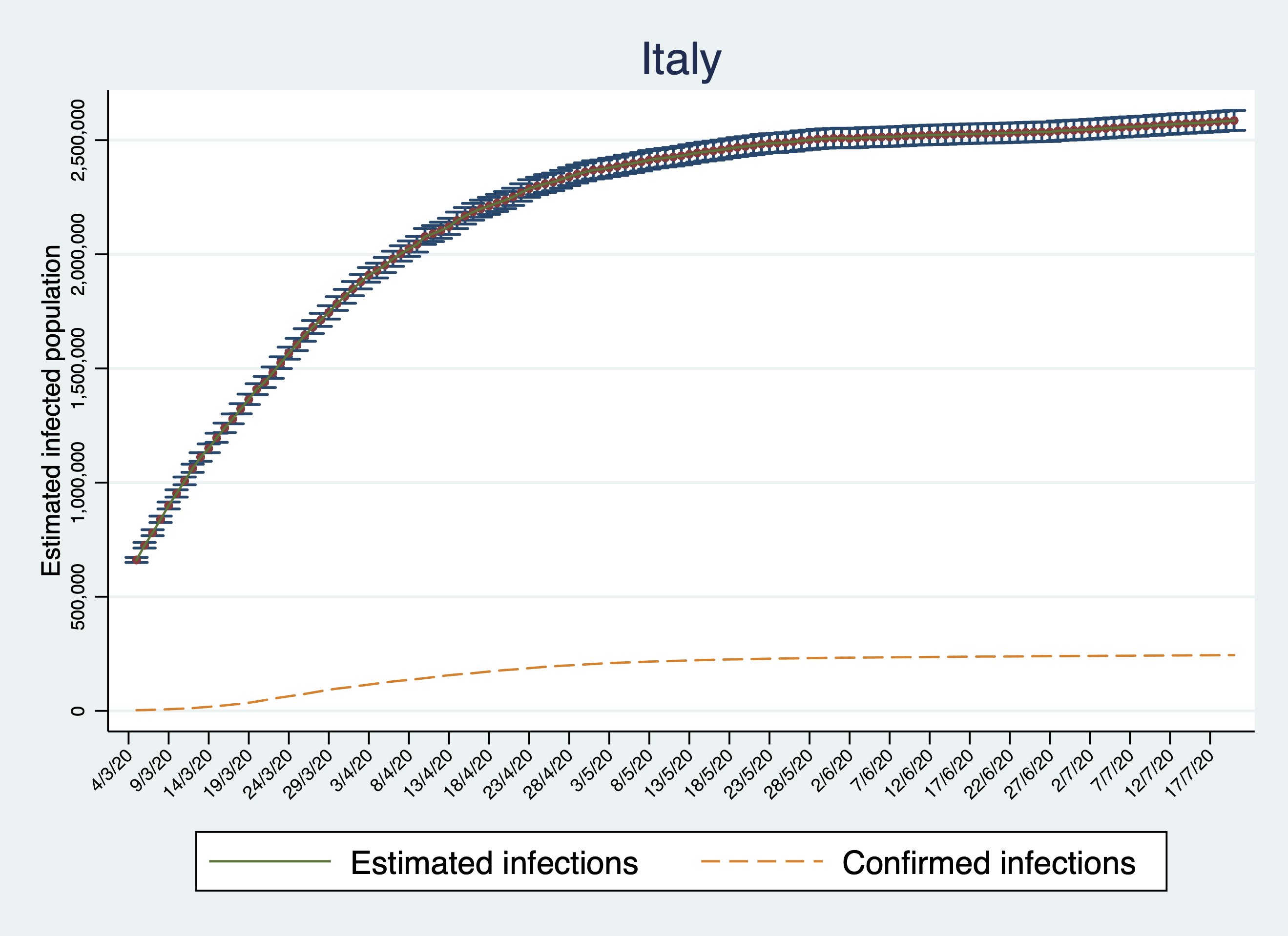

Supplement: Supplementary file 2 [file Data_Sheet_1.ZIP › Country_eni/Italy_20julio.png]

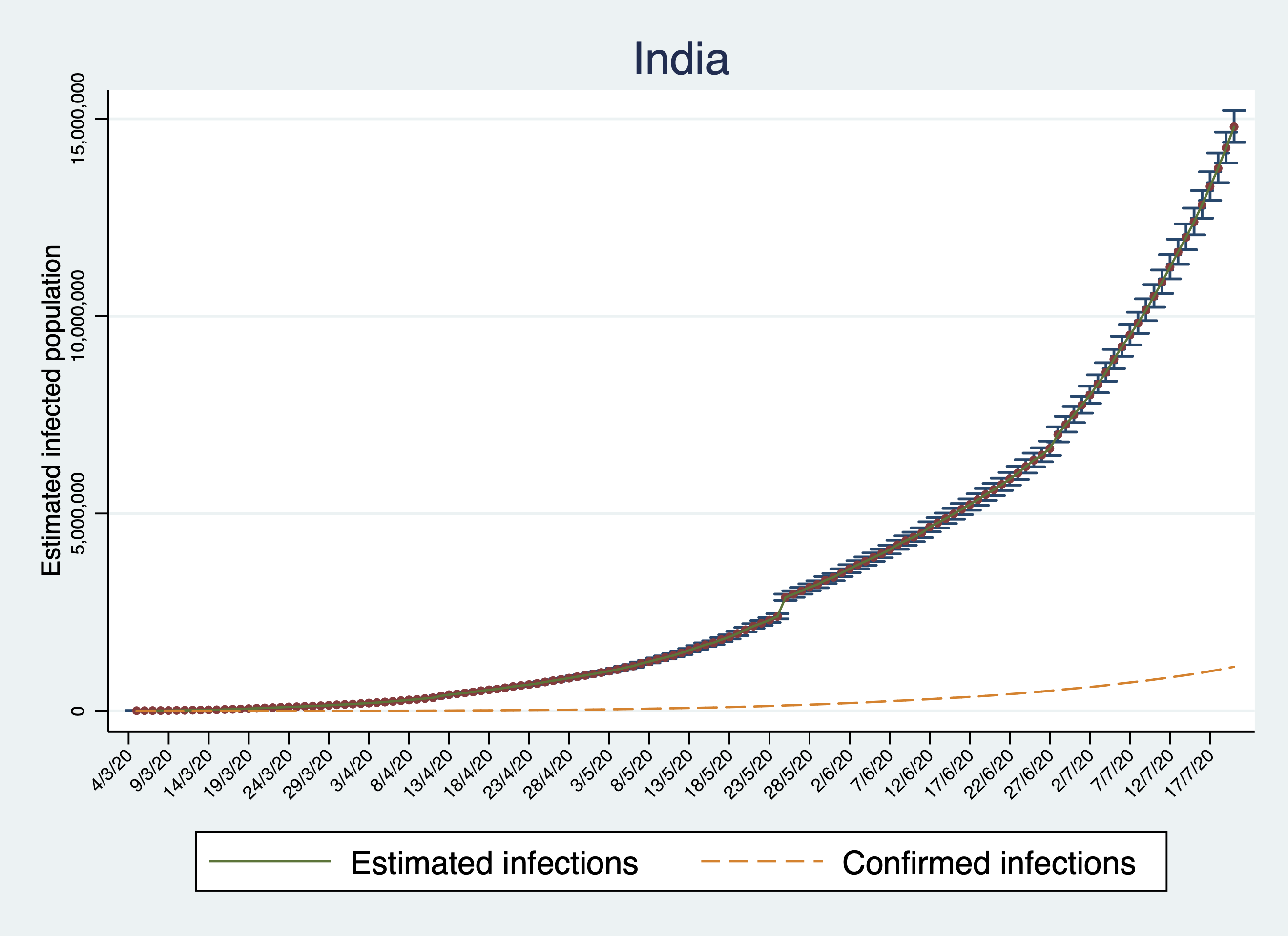

Supplement: Supplementary file 2 [file Data_Sheet_1.ZIP › Country_eni/India_20julio.png]

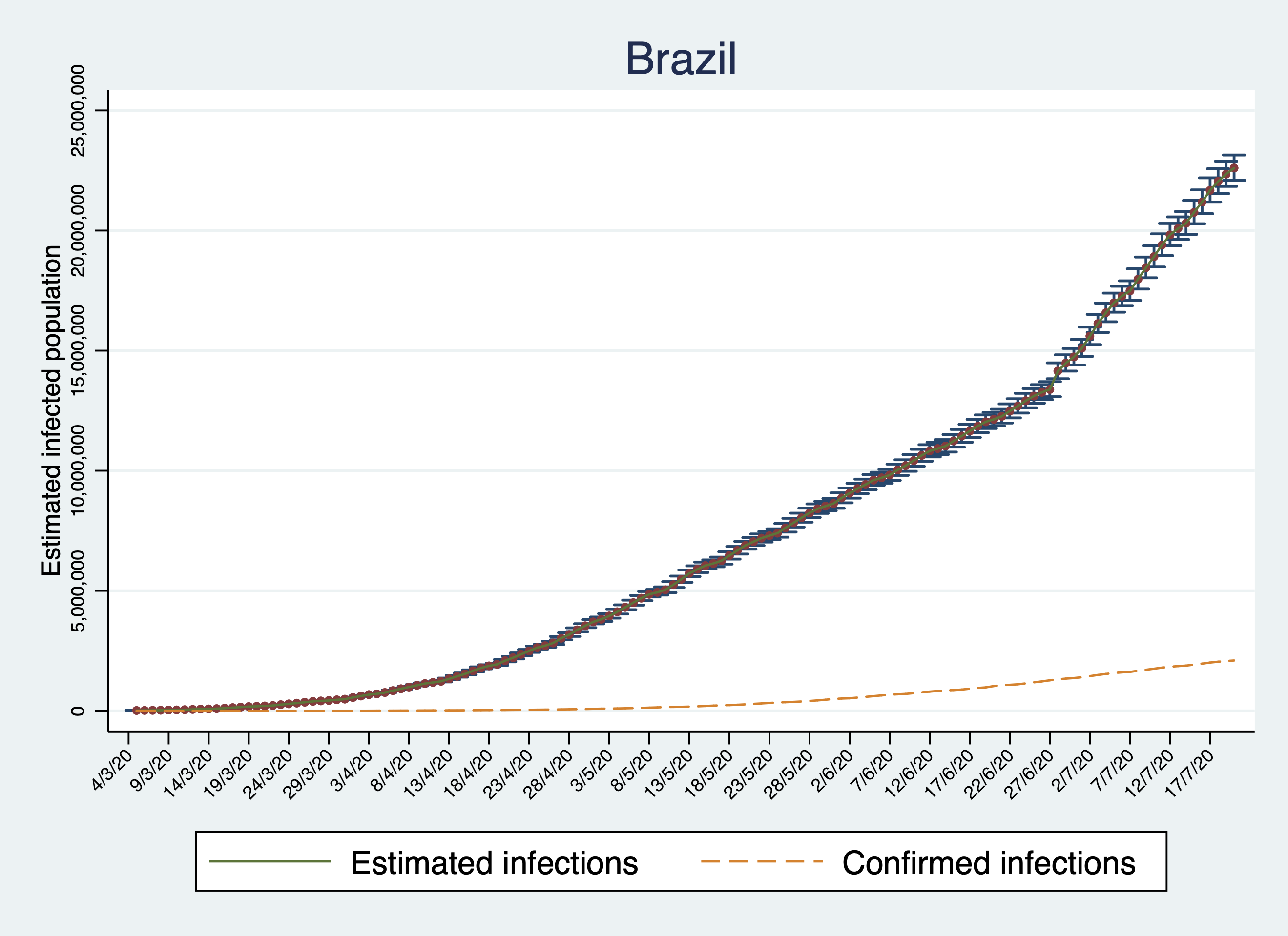

Supplement: Supplementary file 2 [file Data_Sheet_1.ZIP › Country_eni/Brazil_20julio.png]
